# Chemical basis of fluorogenic substrate probe design: revisiting the past to discover the new in biochemistry

**DOI:** 10.1039/d6cb00125d

**Published:** 2026-07-01

**Authors:** Toru Komatsu, Yasuteru Urano

**Affiliations:** a Graduate School of Pharmaceutical Sciences, The University of Tokyo 7-3-1 Hongo, Bunkyo-ku Tokyo 113-0033 Japan tkomatsu@mol.f.u-tokyo.ac.jp; b Graduate School of Medicine, The University of Tokyo 7-3-1 Hongo, Bunkyo-ku Tokyo 113-0033 Japan

## Abstract

Enzyme activity, rather than mere protein abundance, defines the functional state of the proteome. Fluorogenic probes are molecules that are designed to directly report the “activity” of enzymes by fluorescence changes. In this review, we present a comprehensive overview of chemical design strategies for enzyme-activatable fluorogenic substrate probes targeting various classes of enzymes. We discuss the chemical principles required to convert natural substrate reactions into functional molecular sensors that respond to target enzymes with high precision. We also examine how recent advances in these design strategies, together with their integration into innovative analytical platforms, are expanding the impact of enzyme-responsive probes in chemical, biological, and clinical research.

## Abstract 2

Revisiting the chemistry behind classical activity reporter substrates reveals design principles that remain central to modern enzyme-responsive probes. These enduring concepts continue to inspire new technologies for decoding enzymatic activity.

## Graphical abstract

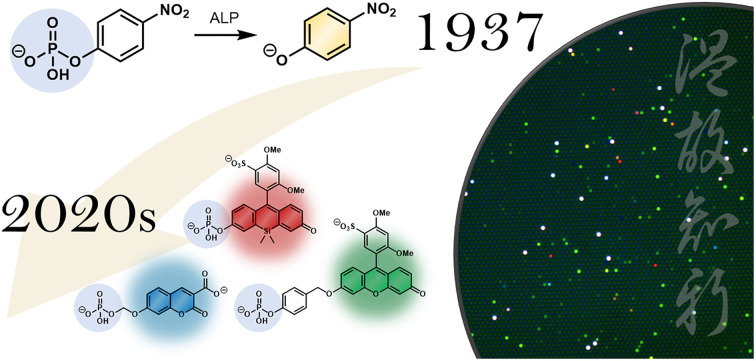

## Introduction

Enzyme-activatable fluorogenic probes have emerged as indispensable tools in modern chemical biology, finding widespread applications in bioimaging, disease diagnosis, and high-throughput screening.^1–6^ The fundamental strength of these probes lies in their ability to translate specific enzymatic activities into detectable optical signals with high sensitivity and spatiotemporal resolution. A wide variety of fluorogenic probes have been developed to monitor enzymatic activities across *in vitro*, *in cellulo*, and *in vivo* systems. Since the “activity” of an enzyme is not a mere reflection of its “amount”^3,4,7–9^—as protein functions in living systems are modulated by various post-translational modifications, protein–protein interactions, and fluctuations in the concentrations of metabolites or cofactors—the layer of enzyme activity can provide unique information that is not directly reflected in genomic, transcriptomic, or proteomic analyses and is more closely related to phenotypic changes. Accordingly, an increasing number of fluorogenic probes are currently being developed to reveal the specific connections between enzymatic activities and phenotypic changes or diseases.^10–13^

**Fig. 1:**
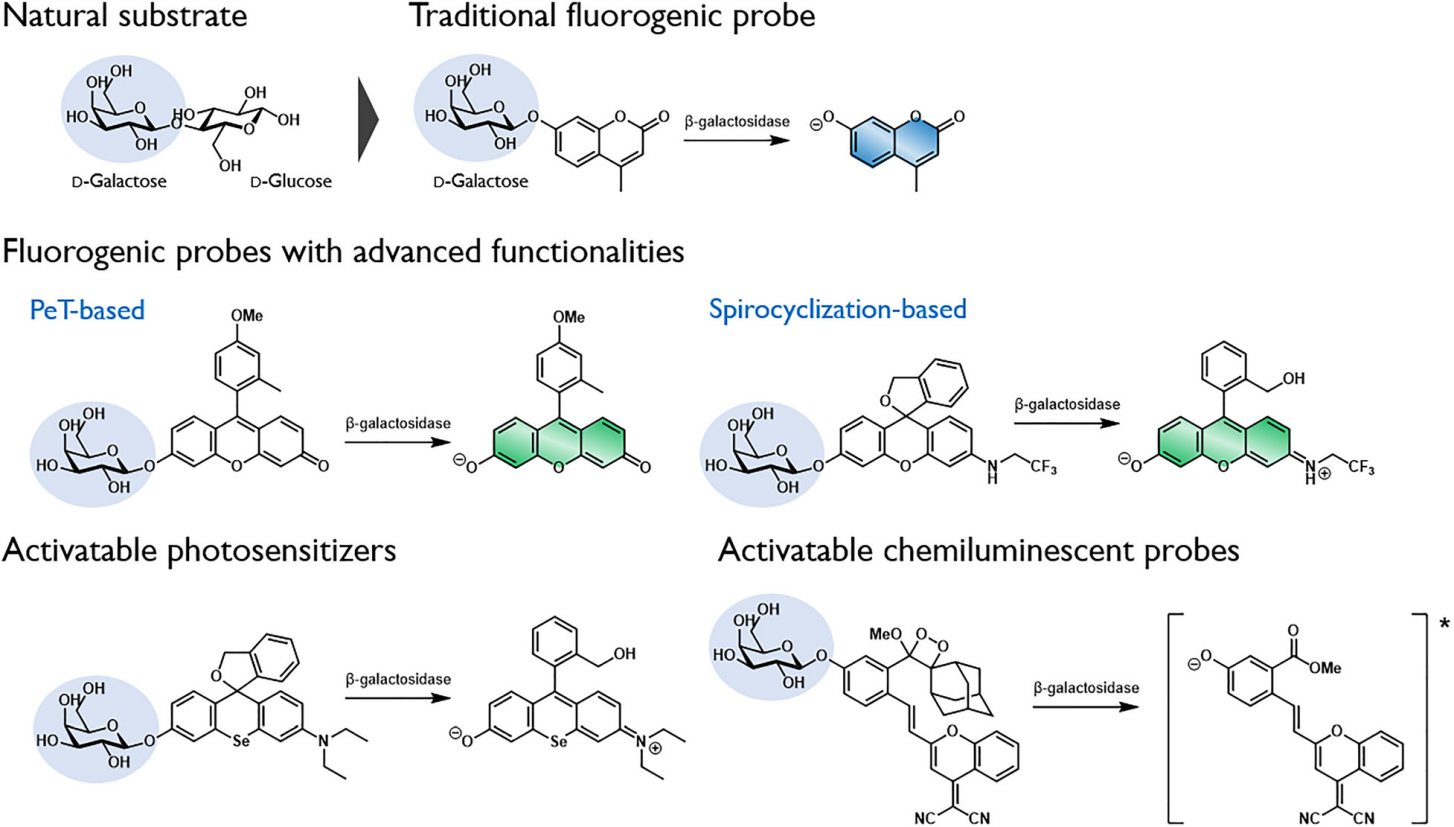
Expansion of chemical tools from the activity of targeted enzyme. Having β-galactosidase as an example, β-galactopyranosyl group, a reaction site, is attached to various functional molecular scaffolds to design chemical tools such as fluorescent probes suitable for live cell imaging,^14,15^ enzymatically activatable photosensitizers,^16^ and chemiluminescent probes.^17^

The general principle of fluorogenic probe design requires that (1) it acts as a faithful mimic of the physiological substrate's reactivity to ensure enzymatic recognition, and (2) it integrates a molecular switch that triggers a change in optical properties upon catalysis (Fig. 1). In addition to these key criteria, probes for practical use in living systems must fulfil (3) sufficient selectivity to report the target enzyme (ensuring the relevance of the signal to the target enzyme) in the system, and (4) proper localisation to meet the target enzyme, which includes appropriate pharmacokinetics for *in vivo* experiments and adequate membrane permeability and cellular distribution for *in cellulo* experiments. In this review, we focus mostly on the state-of-the-art regarding (1) and (2): how one can mimic the substrate structures of enzymes and incorporate signal switching mechanisms using chemical tricks. While numerous studies and reviews have extensively discussed the photophysical mechanisms—such as ICT, PeT, FRET, spirocyclization, and AIE—governing the signal transduction of various fluorophores,^10,12^ there is a distinct lack of literature that integratively discusses how the substrate mimic can be properly designed to enable the targeting of specific enzyme classes across the enzymome. This review focuses mostly on fluorogenic probes, but the design strategies of chemical switches for design enzyme-activatable probes can serve as general principles for other reporter or bio-responsive molecules such as chemiluminescent probes, bioluminescent probes, Raman imaging probes, magnetic resonance probes, or prodrugs that release functional molecules such as drugs and photosensitizing agents.

The core science of designing substrate mimicry originated almost a century ago, but an increasingly diverse repertoire of chemical tricks has since been developed to expand the types of targetable enzymes. Moreover, in contrast to traditional studies targeting purified proteins, attempts to study enzymatic activity in complex biological systems have advanced through integration with various modern techniques, such as various imaging techniques, library-based discovery approaches, data analysis with artificial intelligence (AI), microfluidics, and single-molecule analysis platforms. These advancements have enabled the discovery of novel aspects of phenotype- or disease-related alterations in enzyme functions.

The present categorisation of enzymes largely relies on the enzyme commission (EC) numbers:^18^ (1) oxidoreductases, (2) transferases, (3) hydrolases, (4) lyases, (5) ligases, (6) isomerases, and (7) translocases. Among them, hydrolases occupy the largest class of enzymes, and due to the robust structural changes (bond cleavage) caused by hydrolases, many design strategies have been developed specifically for this enzyme class. Therefore, we primarily aim to provide a guide to the “design rationale” for hydrolases, but we also extend the discussion to how other enzyme classes can be targeted through selected pioneering examples. By bridging the gap between classical biochemistry and modern molecular design, this review serves as a roadmap for developing the next generation of precision chemical tools.

## Fluorogenic probes for hydrolases

### Phosphatses

**Scheme 1:**
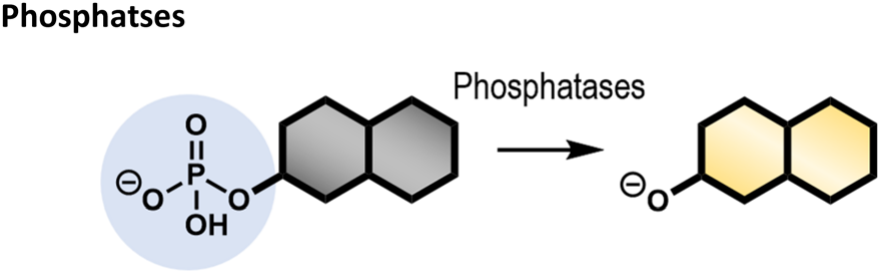
General design of phosphatase probes.

Phosphatases are ubiquitous enzymes that catalyze the hydrolysis of phosphomonoesters into a phosphate ion and a free hydroxyl group (Scheme 1). This class represents the oldest enzyme whose activity was visualised using synthetic substrates in biological samples. In the 1930s, several researchers studied the hydrolysis of phosphate esters in tissue sections and blood samples using various synthetic substrates.^19,20^

**Fig. 2:**
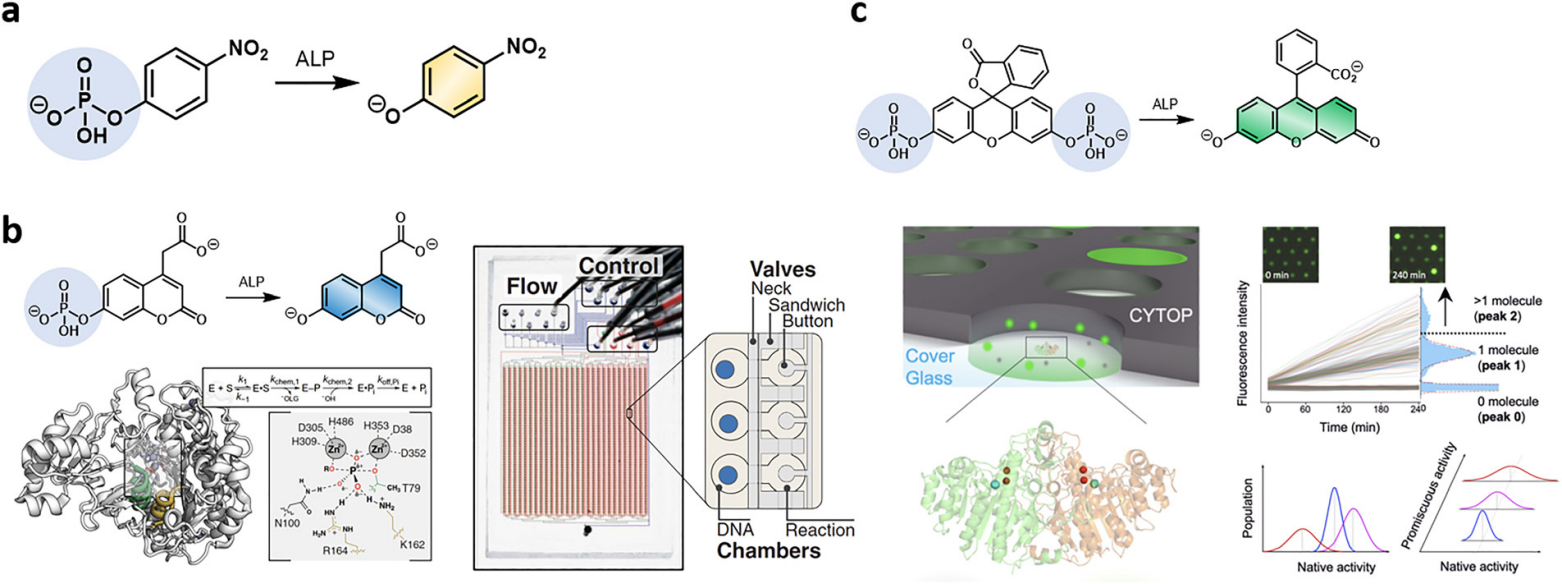
Old and modern applications of fluorescence detection of ALP. (a) Chemical structure of *p*-nitrophenyl phosphate (pNPP) used for detection of ALP activity in blood samples. (b) Platform for simultaneously analysing the activities of mutant ALPs to understand the structure–activity relationships of ALP. Reproduced from ref. 23 with permission from the American Association for the Advancement of Science (AAAS), copyright 2021. (c) Singe-molecule analysis of activities of mutant ALPs to reveal the promiscuity of the activity. Reproduced from ref. 24 with permission from American Chemical Society (ACS), copyright 2023.

Among them, the powerful and still widely employed assay was that based on 4-nitrophenyl phosphate (pNPP) first reported in 1937 by Ohmori.^21^ The design strategy relies on the enzymatic release of 4-nitrophenol (pNP), whose yellow colour of phenolate serves as a direct readout of enzyme activity (Fig. 2a). The classical assay remains used in current clinical diagnostics; the detection of alkaline phosphatase (ALP) activity in blood is a foundational tool for diagnosing liver damage and bone diseases, a practice established in the 1930s–1940s.^22^ Since the phosphate group is a near-universal recognition motif, this “masking group” strategy is commonly applied to the design of fluorogenic probes. Various fluorophores with a phenolic group are masked with a phosphate group, and the change in fluorescence properties upon phosphor-ester-to-phenolate conversion is utilized as the source of fluorogenic readout.

Alkaline phosphatase (ALP) is traditionally used in various biological applications as a reporter enzyme.^25^ Since the activity of ALP can be clearly read out, the enzyme has also been at the centre of attention in studies examining how structural modifications correlate with its activity. Advanced activity readout assays using fluorogenic substrates have been combined with various modern applications to deepen this type of research. Markin *et al.* utilized a high-throughput microfluidic platform (HT-MEK) to characterise over 1000 mutants of an alkaline phosphatase (PafA),^23^ revealing that many mutations throughout the protein compromise catalytic function by promoting a long-lived misfolded state (Fig. 2b). Sakuma *et al.* employed a femtoliter reactor array device to quantitatively analyse molecule-to-molecule functional heterogeneity in over 60 alkaline phosphatase mutants,^24^ discovering that single-point mutations can readily expand functional substates and that this heterogeneity correlates with improved promiscuous activities (Fig. 2c).

Apart from biochemical assays using recombinant enzymes, the challenge of monitoring the activities of endogenous phosphatases remains significant. For example, Liu *et al.* reported *in vivo* imaging of ALP to detect tumour sites,^26^ and Takakusa *et al.* reported live-cell imaging of PTP activities and revealed their modification by oxidative stress.^27^ However, a major challenge in monitoring phosphatase activities in living systems is the diversity of phosphatases—ALP, acid phosphatases, and protein phosphatases—contrasted with only one reaction group available to target the enzyme. ALP serves as the representative member of the phosphate-ester–hydrolyzing class, with ubiquitous expression and relatively non-specific activity toward phosphate esters. Four human genes (ALPL, ALPI, ALPP, ALPP2) were identified for ALP,^28,29^ which play a central role in hard tissue formation (bone mineralization) and intestinal phosphate transport.^30^ Acid phosphatase has five important subtypes (lysosomal, prostatic, erythrocytic, macrophage, and osteoclastic expression). Among them, the biological role of osteoclastic acid phosphatase (OcAP), also called tartrate-resistant acid phosphatase (TRAP), is most prominent due to its role in the regulation of bone metabolism.^31^ Besides these relatively non-specific phosphatases, the larger phosphatase class includes protein phosphatases. They contain various important proteins that play central roles in protein functional regulation by reversing the activity of kinases. For example, PTP1B is a major protein tyrosine phosphatase that regulates signalling pathways downstream of cell-surface receptors, such as the EGFR.^32^ PP2A is a ubiquitous serine/threonine phosphatase that controls a wide array of cellular functions, including metabolism, cell signalling, and the cell cycle.^33,34^ Due to their direct connection with cellular signalling events, their dysregulation contributes to the progression of various diseases, and they serve as good drug targets and biomarkers.^35^ However, targeting specific protein phosphatases using current biochemical assays in complex biological systems remains highly challenging.

**Fig. 3:**
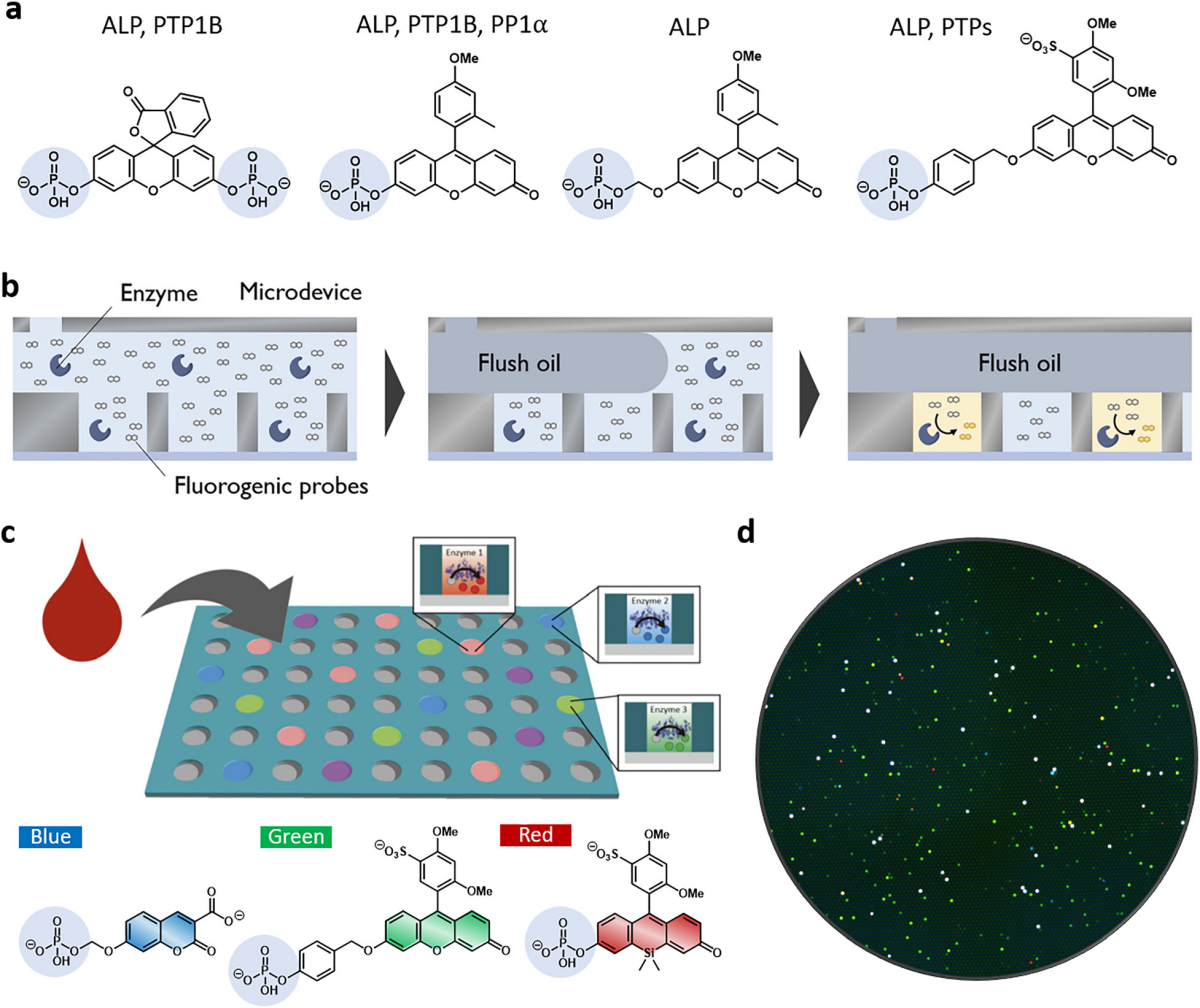
Single-molecule enzyme activity profiling (SEAP) for discriminatively detecting various phosphatases in biological sample. (a) Structures of fluorogenic probes that are designed to diversify the structures attached to phosphate to discriminate different phosphatase species. Names of enzymes indicate the representative target enzymes of probes. (b) The workflow of microdevice-based single-molecule enzyme activity analsyis. Reproduced from ref. 38 with permission from Elsevir, copyright 2026. (c) Concept of SEAP, which uses multi-coloured fluorogenic substrates to distinguish different phosphatase species in biosamples by the different reactivities toward structure-wide, multi-coloured fluorogenic substrates. Reproduced from ref. 37 with permission from AAAS, copyright 2020. (d) Detection of multiple phosphatase species in blood samples of healthy human subject. Reproduced from ref. 7 with permission from ACS, copyright 2025.

One approach to achieving selectivity involves modifying the substructures connecting the phosphate group to the fluorophore. For example, methylene-bridged substrates show a preference toward ALP and PTP1B over PP1α,^36^ while substrates incorporating flexible phenolic structures maintain reactivity toward protein tyrosine phosphatases (PTPs)^37^ (Fig. 3a). Extending this idea, one way to clearly dissect the activities of different subtypes of phosphatases has been proposed by utilizing a single-molecule enzyme activity analysis platform (Fig. 3b). In this format, named single-molecule enzyme activity profiling (SEAP),^7,37^ individual enzyme molecules are captured in femtoliter-sized reaction chambers aligned on a microdevice, and the different phosphatase species are separately detected based on their differential preferences for multi-coloured, structurally distinct fluorogenic substrates (Fig. 3c and d). The study by Sakamoto *et al.* analysed the different phosphatase species in blood samples by discriminatively detecting them with three differently coloured phosphatase substrates.^37^ The assay revealed the presence of diverse phosphatase species in blood samples, containing multiple subtypes of ALPs and PTPs.

Although assignment of the activity species to specific enzyme is a challenging part, this “one-molecule-to-one-activity” analysis is key to discriminatively detecting overlapping phosphatase activities and providing deeper insights into altered enzyme functions in biological and clinical samples than those observed in conventional “multiple-molecules-to-one-activity” analysis performed under bulk conditions.^7,39,40^

### Phosphodiesterases

**Scheme 2:**
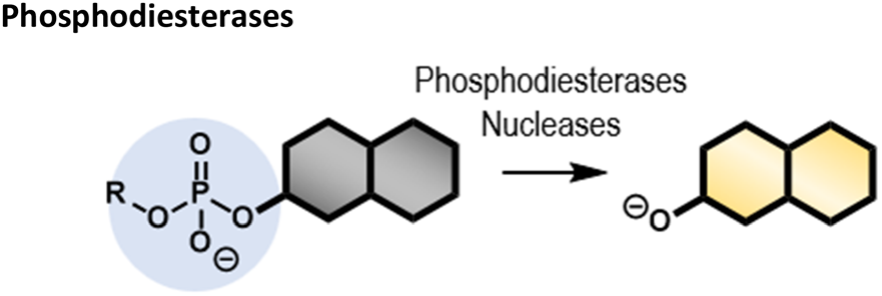
General design of phosphodiesterase probes.

Since phosphate esters are present in many biomolecules, there are diverse enzymes that recognise and metabolize phosphate derivatives.^41^ While phosphate monoesters are metabolized by phosphatases, phosphodiesters can be metabolized by phosphodiesterases (PDEs), such as intracellular PDEs, nucleases, and ectonucleotide pyrophosphatases (ENPPs)^42^ (Scheme 2). From a probe-design perspective, alkylation of the phosphate group of the probe shifts its potential target from phosphatases to phosphodiesterases, and the design diversity and target preference can be optimised by modifying the substituents introduced onto the phosphate group.

**Fig. 4:**
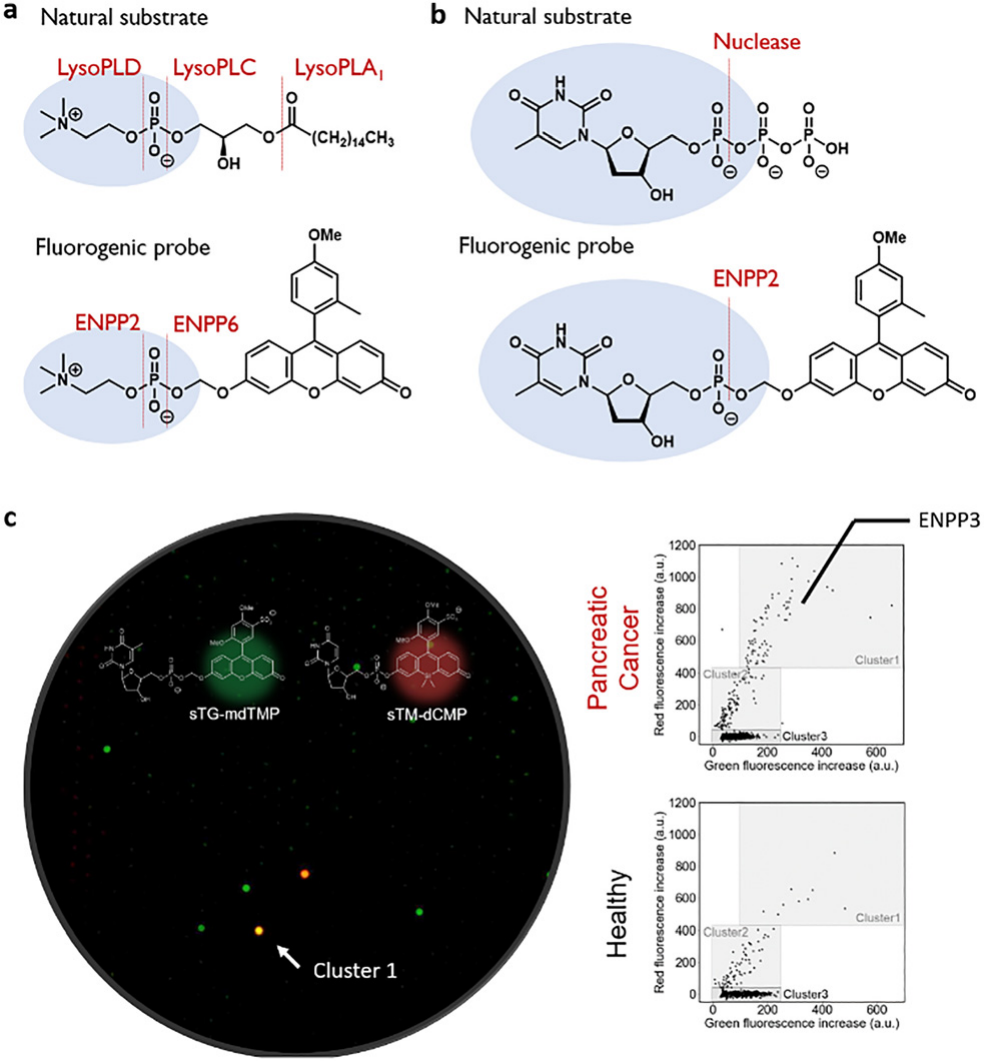
Fluorogenic probes for ENPPs and nucleases. (a) Types of enzyme that use lysophosphatidylcholine (lysoPC) as a natural substrates and the design strategy of fluorogenic probes to have selectivity toward ENPP6 (LysoPLC) over ENPP2 (LysoPLD). (b) Design of fluorogenic probe for ENPP2 targeting its nuclease activity. (c) SEAP platform targeting ENPP1-3 revealed the circulating ENPP3 as a potential biomarker of PDAC.^37^ Reproduced from ref. 37 with permission from AAAS, copyright 2020.

Kawaguchi *et al.* exploited structural differences in how these isoforms recognise their substrates to develop highly selective probes.^43^ A primary example is the development of TG-mPC, a probe specific for ENPP6 (lysophospholipase C; lysoPLC). While both ENPP2 (autotaxin) and ENPP6 act on lysophosphatidylcholine (LPC), they differ in their cleavage sites: ENPP2 exhibits lysoPLD activity, whereas ENPP6 exhibits lysoPLC activity. Accordingly, a specifically designed probe bearing a methylene linker exhibited high selectivity toward ENPP6 over ENPP2 (Fig. 4a), and the fluorogenic probe was used in a high-throughput screening (HTS) assay to characterise various ENPP6 inhibitors.^43^ In a similar fashion, a fluorogenic probe for ENPP2 was developed, and inhibitors of ENPP2, a candidate target for controlling angiogenesis, were identified through HTS.^44^ In the study of ENPP2, a nucleic acid analogue was revealed to be more suitable for monitoring ENPP2 activity than a lysophospholipid analogue (Fig. 4b). This is because ENPP2 also possesses nuclease activity, and the result demonstrates the applicability of this phosphodiester probing strategy for designing fluorogenic probes for various nucleases. As an extension, a fluorogenic probe targeting the tumour suppressor enzyme fragile histidine triad (FHIT) protein was also developed.^45^

If one seeks to monitor the activity of ENPP family members and related phosphodiesterases and nucleases in biological systems, a challenge similar to that encountered with phosphatases arises: the need to discriminatively detect closely related and overlapping activities. While substrate specificity can be tuned by minor modifications of the R group, it remains challenging to design a completely independent reporter molecule for each ENPP family member. In such situations, the SEAP platform is also suited to discriminatively detect individual ENPP activities, as in the case of phosphatases^37^ (Fig. 4c). Since common probe-design strategies can be applied, SEAP assays were constructed for ENPPs, revealing the presence of active ENPP1 and ENPP3 in circulation. The presence of these enzymes in blood samples had not previously been recognised due to their low abundance in circulation. Interestingly, the study revealed an alteration of ENPP3 activity in blood samples from pancreatic adenocarcinoma (PDAC) patients^37^ (Fig. 4c). The biological basis of this phenomenon remains unclear; however, ENPP3 was recently identified as a major regulator of extracellular cGAMP and has been reported to exhibit altered expression in cancer cells.^46^ This raises the possibility that increased ENPP3 levels in the bloodstream may be correlated with these observations. A recent study also revealed the presence of ENPP activity in pancreatic juice,^47^ so it is also possible that this finding reflects the leakage of pancreatic enzymes into the bloodstream due to obstruction of the pancreatic duct in PDAC.

### Glycosidases

**Scheme 3:**
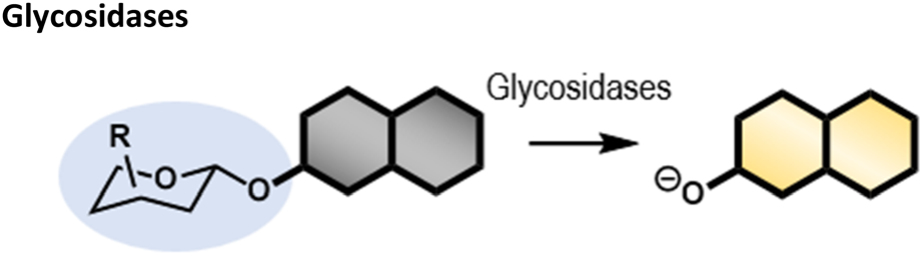
General design of glycosidase probes.

**Fig. 5:**
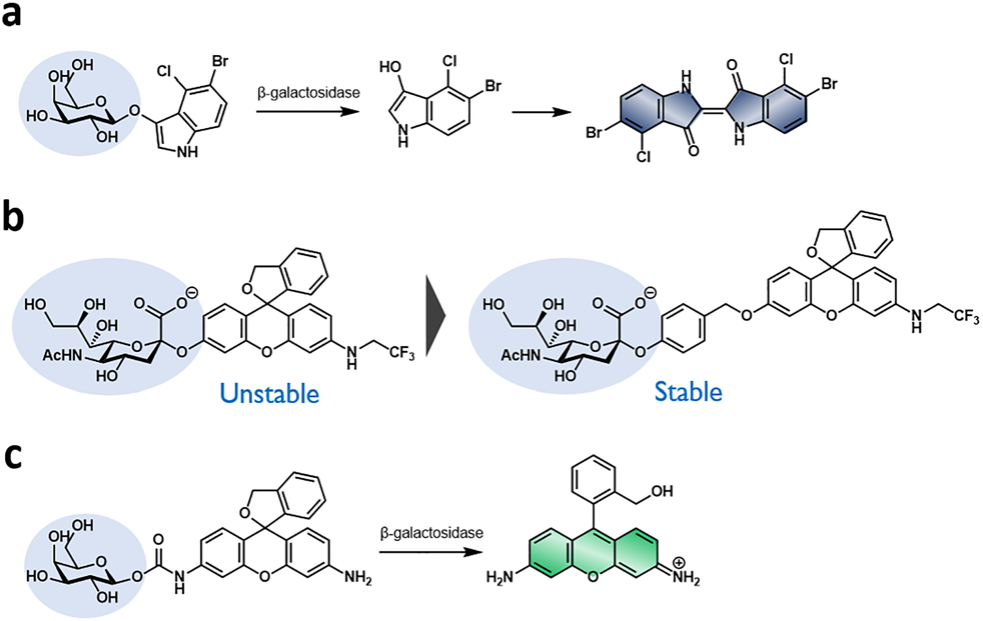
Traditional colorimetric probe and extension of the reaction site design. (a) Chemical reaction of X-gal. (b) Design strategy of fluorogenic probe for glycosidase using PHBA linker.^48^ (c) Glycosidase probes with carbamate reaction site.^49^

The “masking group” approach for phenolate-type fluorophores can also be applied to glycosidases, another traditional enzyme class whose activity has long been monitored in biological research (Scheme 3).^52–54^ One of the traditional reporter substrates for glycosidases is X-gal, which targets the lacZ protein, an *E. coli*–derived β-galactosidase (Fig. 5a). As represented by this structure, masking a phenol group with a glycosidic bond has been the major design strategy throughout the history of glycosidase probes.^54^ For longer-wavelength probes containing multi-aromatic rings, catalytic activity may be compromised, and for acidic phenols, some glycosides are not sufficiently stable, resulting in background cleavage. To address these issues, self-cleavable linkers, such as *p*-hydroxybenzyl alcohol (PHBA) derivatives, have been introduced; cleavage of the glycoside leads to 1,6-elimination, which subsequently cleaves a phenol ether or carbamate, releasing phenol-type or aniline-type fluorophores^48,55^ (Fig. 5b).

Glycosyl carbamates can also act as substrates for glycosidases, enabling the design of various functional molecules based on this strategy^49^ (Fig. 5c). The major use of X-gal and related compounds in earlier research periods was to perform reporter gene assays, in which the expression of lacZ is read out by colorimetric or fluorescent signals. Reflecting this early research focus, those studies primarily targeted glycosidase activities from exogenous sources.^14,56^ This was partially due to the low detection sensitivity of traditional assays; however, recent advances in fluorogenic probe designs have enabled the sensitive detection of endogenous glycosidases in living mammalian cells, revealing their importance in various biological events and diseases.^53,54^

**Fig. 6:**
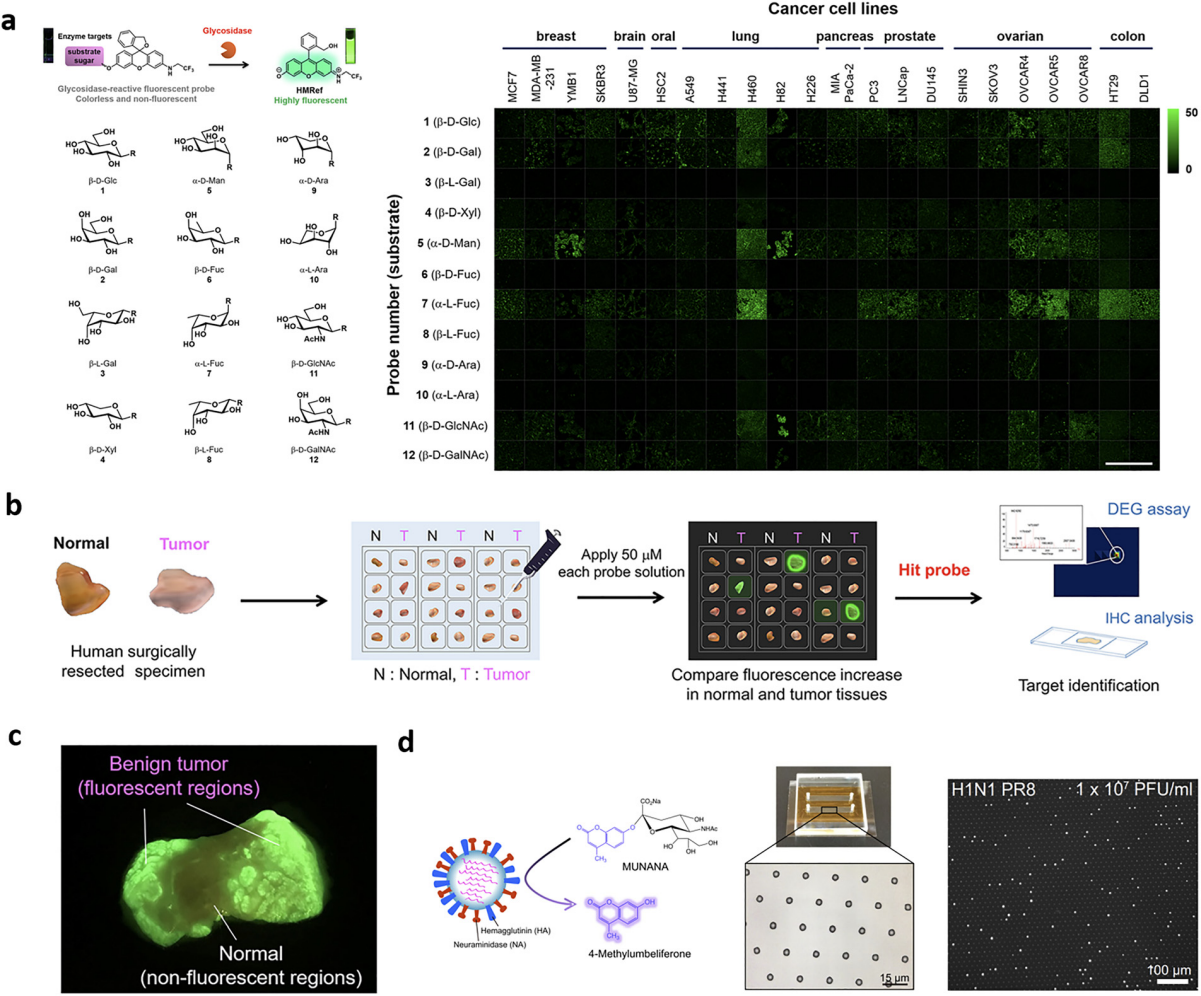
Recent applications of monitoring endogenous glycosidase activities in living systems. (a) Global analysis of glycosidase activities using the library of fluorogenic probes for glycosidases. Reproduced from ref. 50 with permission from ACS, copyright 2020. (b) The scheme of resected specimen-based screening to identify tumour-specific alterations of glycosidase activity. Reproduced from ref. 50 with permission from ACS, copyright 2020. (c) Detection of altered α-mannosidase activity in breast tumour specimen. Reproduced from ref. 50 with permission from ACS, copyright 2020. (d) Use of sialidase activity to detect single viral particle for ultra-sensitive detection of influenza virus without the use of antibodies. Reproduced from ref. 51 with permission from Springer Nature, copyright 2019.

Fujita *et al.* studied the activities of various glycosidases in numerous mammalian cells using a library of 12 fluorescent probes, which revealed the major activities were observed for β-glucosidase, β-galactosidase, α-l-fucosidase, α-mannosidase, and hexosaminidase^50^ (Fig. 6a). Although the observed activity might be partially influenced by the fluorophore structure, this study provides a general roadmap for designing the substrate moieties of glycosidase-targeting molecules that function in live-cell assays. The five activities observed are reasonable, as these glycosidic bonds correspond to those found in the carbohydrate chains of glycoproteins and glycolipids; this correlation implies that the major role of these glycosidases is the catabolism of these carbohydrate chains. Comprehensive glycosidase activity profiling was subsequently applied to clinical specimens to identify activity-based biomarkers. Probes exhibiting differential signals between normal and tumor tissues were selected as candidates reflecting disease-associated alterations in glycosidase activity (Fig. 6b). This line of research revealed altered activities of specific glycosidases in various cancers: α-mannosidase (specifically the subtype MAN2C1) in breast cancer cells,^50^ hexosaminidase in colorectal cancer cells,^57^ and α-l-fucosidase activity in liver cancer cells^58^ (Fig. 6c).

Besides the prominent activity alterations in cancer cells, the altered activity of β-galactosidase is well known to be correlated with the cellular senescence. The senescent cells show elevated activity of lysosomal β-galactosidase (SA-β-gal),^59^ and β-galactosidase-responsive fluorogenic probes are useful for staining senescent cells within complex cellular population.^56,60^

Another interesting target is sialidase, also known as neuraminidase, which catalyzes the hydrolysis of the glycosidic bond of sialic acid.^61^ Since sialic acid plays an important role in controlling the properties of carbohydrates by adding negative charges, its metabolism is related to various cellular events and diseases, such as cell–cell recognition and immune evasion.^62^ In mammals, dysregulation of these enzymes is closely associated with various pathologies including cancer and sialidosis,^63^ whereas in viruses such as influenza, sialidase activity is vital for promoting the release of new virions from host cells. Therefore, viral sialidase is an important target of antiviral (anti-influenza) drugs. Visualizing sialidase activity is valuable for the sensitive diagnosis of viral infections and the quantitative assessment of antiviral drug efficacy. Tabata *et al.* established the digital influenza virus counting (DIViC) method, which enables the detection of individual virions by stochastically confining them in femtoliter microreactors for a fluorogenic neuraminidase assay^51,64^ (Fig. 6d). This ultra-sensitive, antibody-free approach achieves a detection limit more than 1000 times superior to commercial rapid diagnostic kits, offering a robust platform for early-stage infection diagnosis and quantitative analysis of viral heterogeneity.

### Proteases

Proteases are enzymes that hydrolyze peptide bonds. While peptide bonds are stable under most chemical reaction conditions, these enzymes cleave them through diverse catalytic mechanisms. Proteases play indispensable roles in a wide range of biological processes, including intracellular protein homeostasis, signal transduction, tissue remodelling, and the regulation of diverse physiological cascades.^65,66^ Representative examples include caspases, which are central to programmed cell death; matrix metalloproteinases (MMPs), which facilitate extracellular matrix (ECM) remodelling; factor X and thrombin, key components of the coagulation system; and the proteasome, which governs amino acid recycling and antigen presentation.

**Fig. 7:**
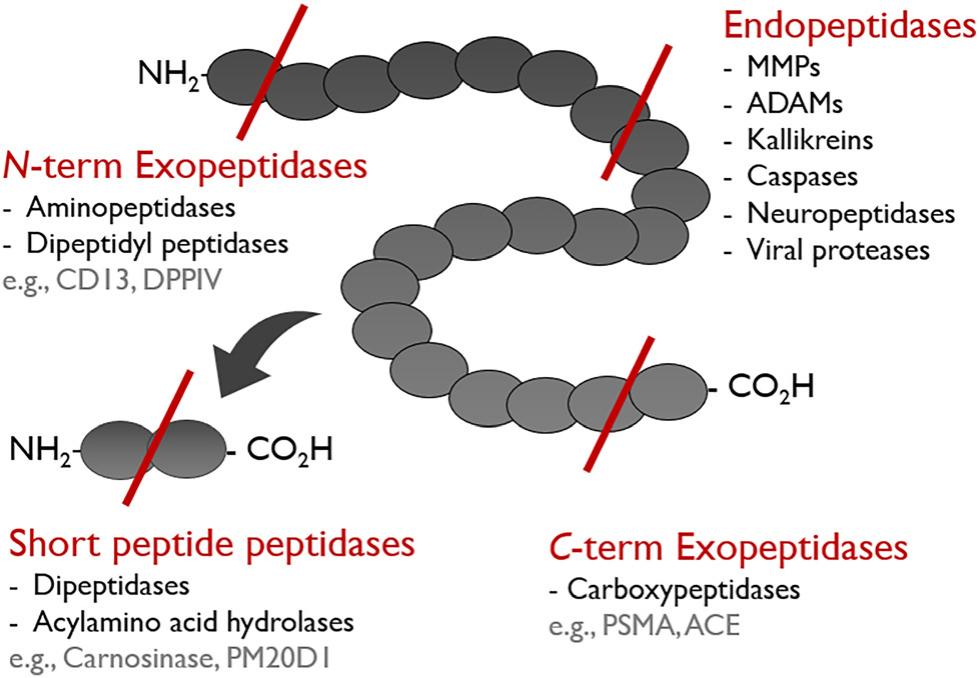
Types of proteases and peptidases. Representative enzymes in the class are shown.

These enzymes are generally classified into two major subclasses—exopeptidases and endopeptidases—based on their mode of substrate recognition and cleavage. Exopeptidases recognise terminal regions of peptides and cleave them sequentially, releasing one or two amino acids at a time. Below, we discuss representative design strategies for targeting each class of proteases (Fig. 7).

### Aminopeptidase

Aminopeptidases recognise the first amino acid at the N-terminus, and dipeptidyl peptidases recognise the first two amino acids at the N-terminus. In both cases, peptides with a free N-terminal residue are highly preferred, indicating that these enzymes recognise the N-terminal cationic amino group. In contrast, another class of enzymes, acylamino acid-releasing enzymes, recognises *N*-acylated amino acid N-termini.^67–69^ The major roles of these exopeptidases include the processing of bioactive peptides to control their signalling and the degradation of intracellular short peptides for antigen presentation or recycling to nutrients.

**Scheme 4:**
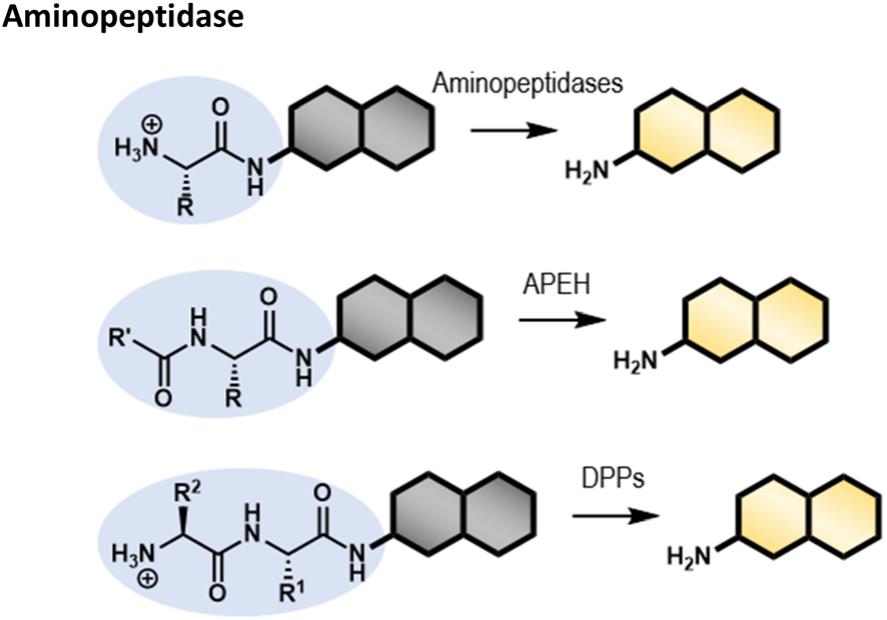
General design of aminopeptidase/dipeptidyl peptidase probes.

**Fig. 8:**
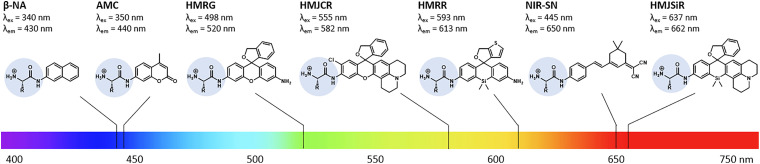
Various scaffolds for the design of fluorogenic probes for aminopeptidases.^70–72^*λ*_ex_ represents the excitation wavelengths, and *λ*_em_ represents the emission wavelengths. β-NA = β-naphtylamine, AMC = 7-amino-4-methylcoumarin, HMRG = hydroxymethyl rhodamine green, HMRR = hydroxymethyl rhodamine rouge.

Like other reporter substrates, early development of aminopeptidase assays relied on colorimetric substrates, most notably derivatives of *p*-nitroaniline (pNA) (Scheme 4). These substrates were designed by modifying specific amino acids into *p*-nitroanilide derivatives, so that the enzymatic reaction results in the release of the yellow-coloured pNA. This “anilide-to-aniline” strategy remains a cornerstone of modern assays, having evolved into fluorogenic sensing platforms utilizing β-naphthylamine, 7-amino-4-methylcoumarin (AMC), rhodamines, and rhodols,^1^ and probes with linkers for aza-quinone methide-forming 1,6-elimination reaction (Fig. 8). While these platforms vary in optical properties, their substrate preference is generally discussed in the context of the P1 amino acid and the tolerance of the P1′ leaving group.

**Fig. 9:**
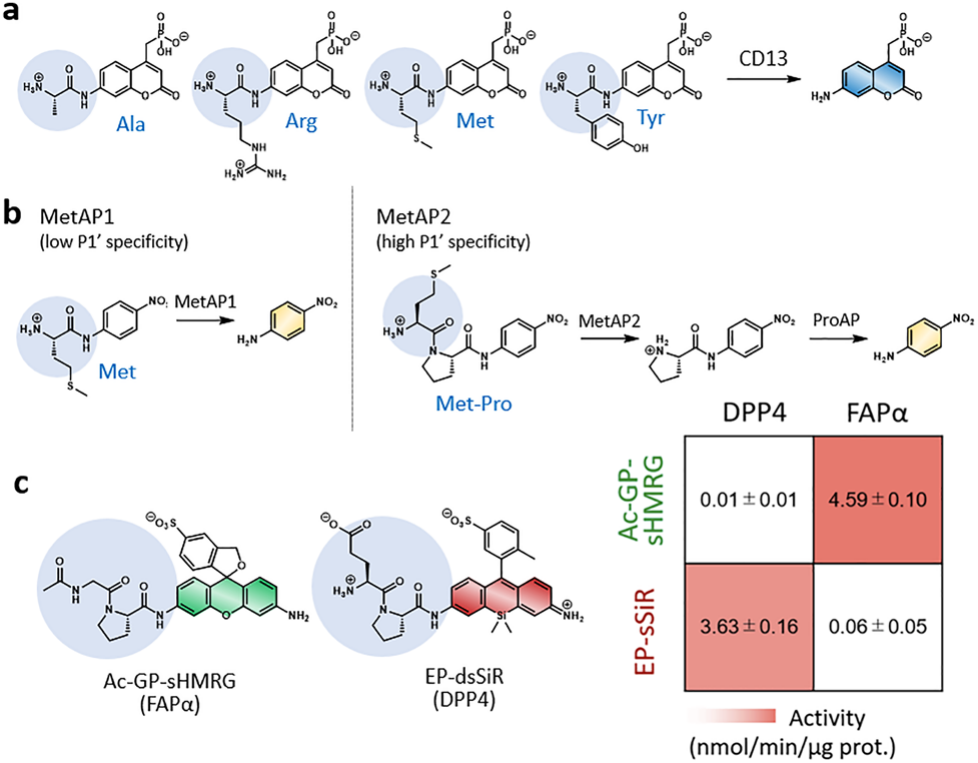
Fluorogenic probes for aminopeptidases. (a) Fluorogenic probes that react with CD13.^75^ (b) Fluorogenic probes for MetAPs. (c) Fluorogenic probes for FAPα and DPP4 and their orthogonality toward target enzymes. Reproduced from ref. 38 with permission from Elsevir, copyright 2026.

A representative and extensively studied example is aminopeptidase N (APN, also known as CD13), a Zn^2+^-dependent “moonlighting” ectoenzyme.^73,74^ CD13 plays a pivotal role in angiogenesis and tumour progression and is widely investigated as a potential drug target and diagnostic biomarker. Originally identified as an alanyl aminopeptidase, CD13 exhibits broad P1 tolerance, accepting various residues such as Arg, Met, and Tyr^75^ (Fig. 9a). There are also aminopeptidases whose P1 or P1′ recognition is highly specific. Methionine aminopeptidases (MetAPs) show stringent specificity for Met at the P1 position, reflecting their essential role in the co-translational processing of nascent proteins.^76^ In humans, two isoforms exist, MetAP1 and MetAP2, both of which are functionally critical for the precise maturation of ribosome-synthesized proteins.^77^ Interestingly, while MetAP1 can tolerate various fluorophores as P1′ mimics, MetAP2 strictly requires a peptide-like environment and frequently fails to recognise substrates in which a bulky fluorophore is directly attached at the P1′ position.^78^ This structural intolerance at the P1′ site poses a significant challenge, necessitating the use of linkers or self-immolative spacers to satisfy the enzyme's recognition requirements. One solution often applied is the use of a coupled assay. For example, Met-Pro is attached to the chromophore pNA, and cleavage by MetAP2 results in the formation of Pro-pNA, which can subsequently be cleaved in the presence of prolyl aminopeptidase to cause the colorimetric product^78^ (Fig. 9b).

**Fig. 10:**
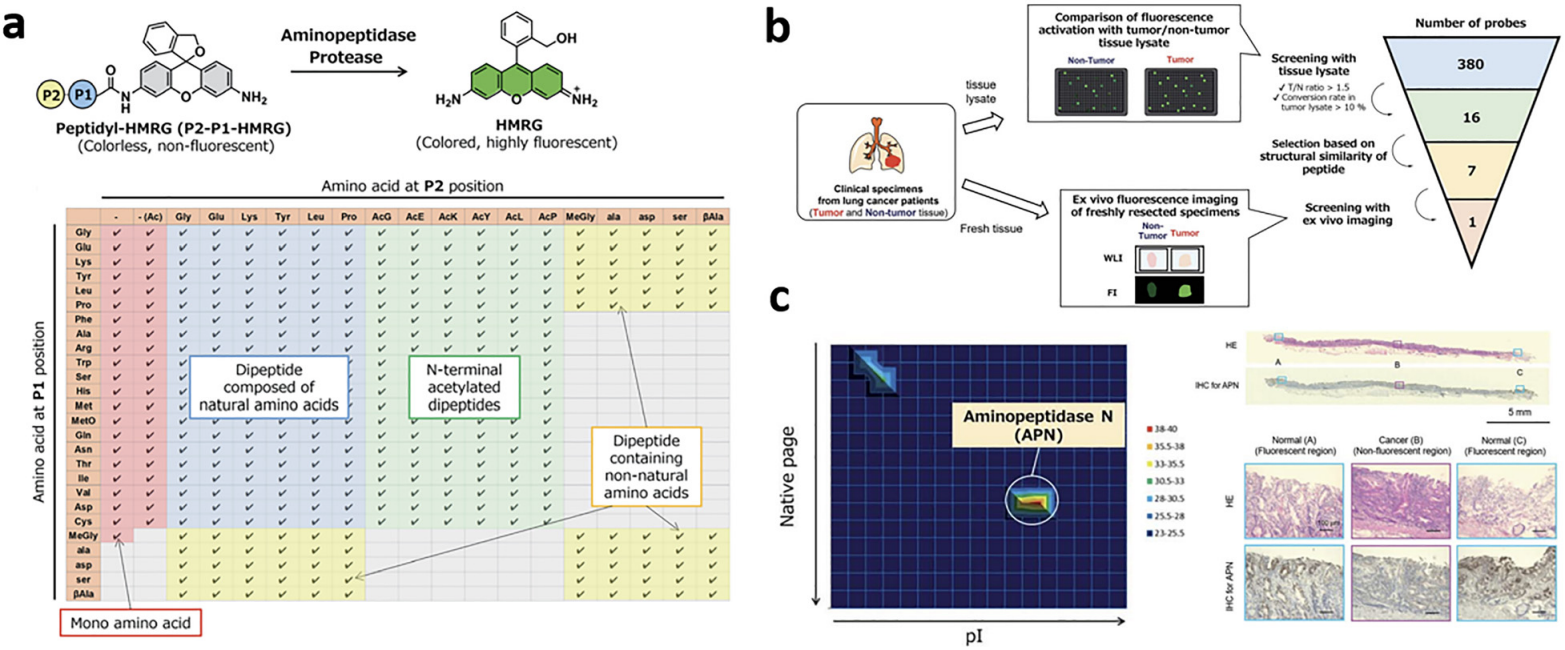
Screening of the tumour-specific activity alteration of aminopeptidases. Reproduced from ref. 79 with permission from Royal Society of Chemistry (RSC), copyright 2022. (a) Fluorogenic probe library targeting various aminopeptidases and dipeptidyl peptidases. (b) Scheme of preparing for the library of fluorogenic probes to identify the aminopeptidases with the altered activities in tumour site. (c) Identification of altered activity of aminopeptidase N (APN, CD13) in the breast cancer cells using diced electrophoresis gel (DEG) assay.^68,80–82^

A similar design principles applies to dipeptidyl peptidases. While the majority of human DPPs, such as the S9B family (DPP4, 8, 9, and fibroblast activating protein α (FAPα)), are characterised as serine hydrolases operating at physiological pH, there are other classes of DPPs that function in lysosomes, such as DPP1 (cathepsin C) and DPP2. For these peptidases, the “anilide-to-aniline” strategy is also employed as the core design principle, but two amino acids are modified to serve as the reaction site (Fig. 9c and 10a). For example, DPP4 prefers the XP or XA motif, where X (the P2 amino acid) can accommodate various amino acids and can be used to modulate physical characteristics or selectivity among the enzyme class.

For example, FAPα and DPP4 are closely related proteins, sharing approximately 50% overall amino acid homology. Both enzymes recognise P1 Pro, but FAPα does not recognise substrates containing Glu at the P2 position.^38,83,84^ Conversely, FAPα also possesses proline endopeptidase-like activity;^38,49^ thus, the Glu-Pro sequence shows specificity toward DPP4, while the Ac-Gly-Pro sequence shows preference toward FAPα, allowing these two enzymes to be separately detected through appropriate amino acid sequences (Fig. 9c). Similarly, differentiation of DPP4 and DPP9 was achieved using Glu-Pro; Gly-Pro or Ser-Pro sequences were cleaved by DPP9, whereas selectivity increased in the case of Glu-Pro.^85^

While the full correlation between P1 (and P1′) amino acids and distinct classes of aminopeptidases has not been fully characterised, experiments using libraries of fluorogenic probes modified with dipeptides as reaction sites are revealing which types of reactive groups are best suited for studying the activities of specific aminopeptidases and dipeptidyl peptidases in complex biological samples (Fig. 10a and b). This study design is based on a top-down omics-type approach (enzymomics),^86^ in which the library is screened in biological samples of interest, and once specific activity is discovered, the responsible enzyme is characterised by biochemical assays such as two-dimensional electrophoresis gel analysis.^67,68,79,80,82^ Through this approach, researchers have revealed altered activities of DPP4 in esophageal, pancreatic, and lung tumours;^84^ leucine aminopeptidase (LAP) in breast and ovarian tumours; puromycin-sensitive aminopeptidase (PSA) in lung and bile duct tumours; and aminopeptidase N (APN, CD13) in stomach tumours^79^ (Fig. 10c).

One representative enzyme whose alteration became evident through activity-based analysis is γ-glutamyl transferase (GGT).^87–89^ Although this enzyme is categorized as a transferase that transfers γ-glutamyl groups to amino groups, in the absence of an acceptor it also exhibits aminopeptidase-like activity that cleaves γ-glutamyl amides. Diverse GGT activity-reporting probes have been reported, and studies in various tumour tissues have revealed alterations in breast, lung, liver, head and neck, and thymus tumours.^87–89^ The reason why GGT activity is altered in many tumours is that the enzyme plays a central role in maintaining cellular redox equilibrium through glutathione (GSH) metabolism, thereby promoting tumour proliferation and conferring resistance against antitumour agents.^90^ Therefore, GGT activity is highly condition-dependent and can be dynamically induced by the tumour microenvironment.

**Fig. 11:**
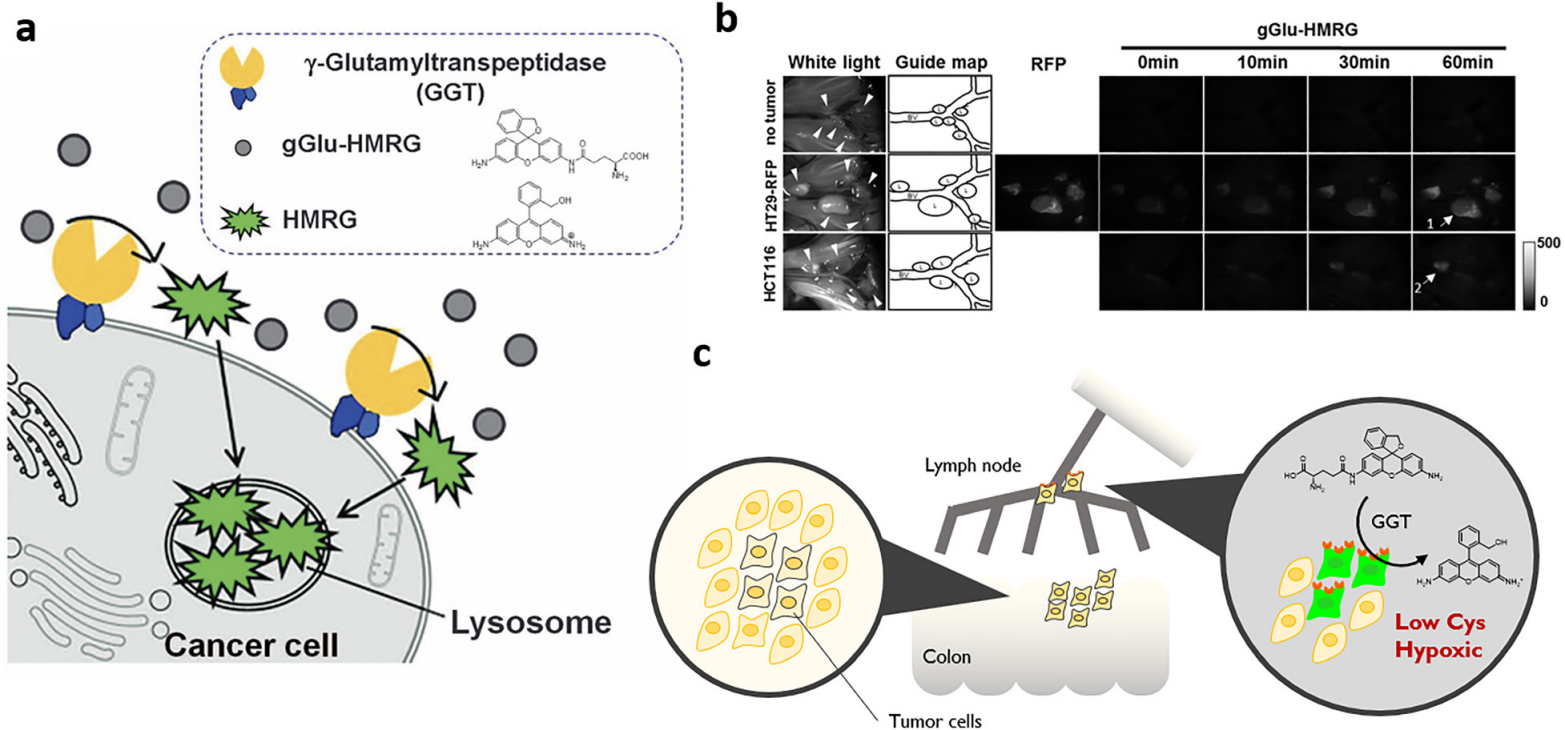
Tumour-specific alteration of GGT activity. (a) The schematic view of tumour imaging using GGT-activatable fluorogenic probes. Reproduced from ref. 89 with permission from AAAS, copyright 2011. (b) Increased GGT activity of colorectal cancer cells in the metastatic lymph node (mLN). Reproduced from ref. 90 with permission from Springer Nature, copyright 2018. (c) Schematic view describing the increased GGT activity in mLN.

For example, Kubo *et al.* demonstrated that while GGT-activatable probes were ineffective for primary colorectal cancer (CRC) due to low basal activity, they successfully visualised metastatic lymph nodes (mLNs). This selective upregulation in mLNs is driven by induction of GGT under conditions of hypoxia and low nutritional status (such as methionine/cysteine-free environments), which trigger oxidative stress within the lymph node microenvironment^90^ (Fig. 11b and c). These findings highlight GGT as a sensitive biomarker for identifying metastatic lesions that have adapted to harsh physiological conditions.

**Fig. 12:**
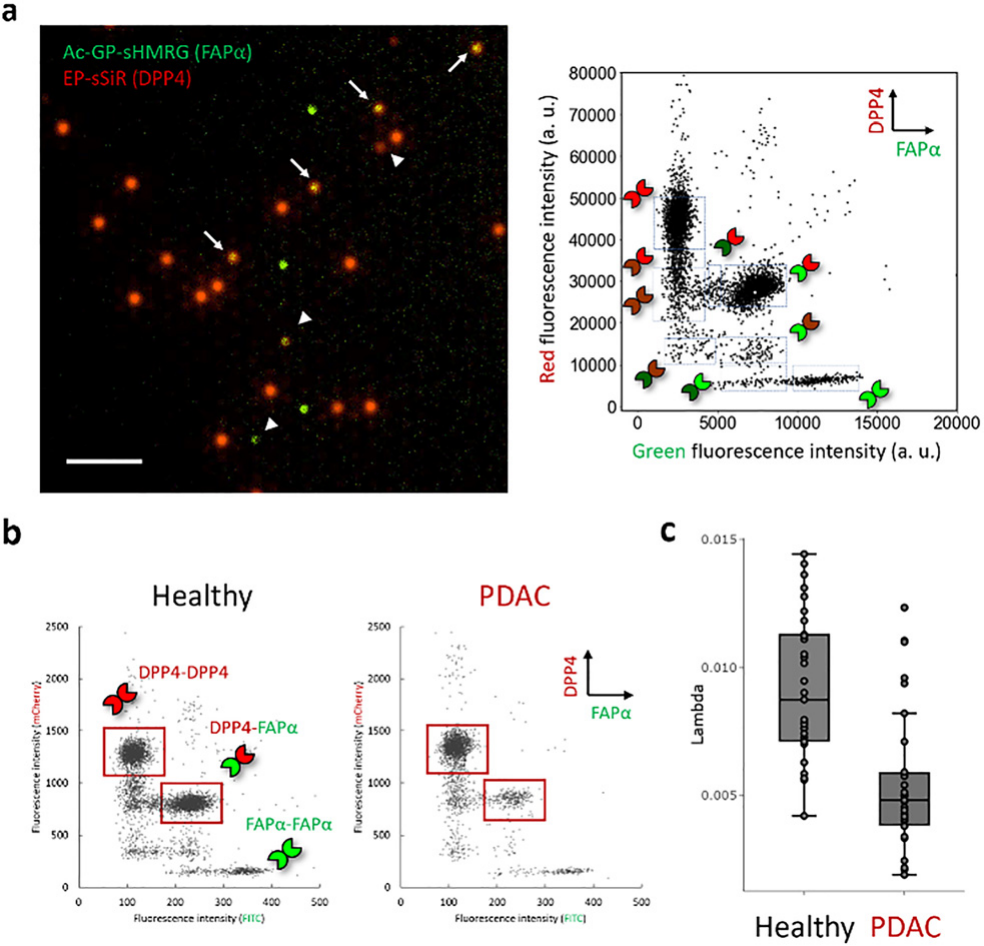
Single-molecule analysis to reveal the PDAC-specific biomarker species having both DPP4 and FAPα activity. Reproduced from ref. 38 with permission from Elsevir, copyright 2026. (a) Fluorescence spots of single-molecule enzyme activity analysis of blood samples using fluorogenic probes for DPP4 (red) and FAPα (green). (b) Alteration of the patterns of single-molecule enzyme activities in blood samples of patients with PDAC.

Recently, the concept of enzymomics has been extended to single-molecule enzyme activity assays.^38,75^ Construction of a library of single-molecule enzyme assays enabled the detection of various enzyme activities present in plasma samples, and alterations of DPP4 and CD13 were detected in blood samples from pancreatic tumour patients^38,75^ (Fig. 12a). Interestingly, the assay revealed colocalisation of activity species bearing DPP4 and FAPα, indicating the presence of a heterodimer of DPP4 and FAPα in blood samples.^38^ This species is downregulated in blood samples from PDAC patients and therefore may serve as a source of activity-based diagnostics (Fig. 12b).

### Endopeptidases

While aminopeptidases and dipeptidyl peptidases act on the N-termini of peptide chains, endopeptidases cleave peptide bonds within the polypeptide sequence. The human genome encodes a vast array of endopeptidases, including more than 400 members such as MMPs, cathepsins, and caspases.^66^ For example, enzymes with trypsin-like activities prefer basic P1 amino acids (Arg and Lys), chymotrypsin-like activities prefer aromatic P1 amino acids (Tyr, Phe, and Trp), elastase-like activities prefer small hydrophobic P1 amino acids (Leu, Val, Ile, and Ala), and caspase-like activities prefer acidic P1 amino acids (Glu and Asp).^91^ While the “anilide-to-aniline” strategy using small fluorophores can be applied to endopeptidases that tolerate a bulky P1′ leaving group,^92,93^ endopeptidases generally exhibit distinct preferences for specific residues on the C-terminal side (P1′, P2′, *etc.*) for substrate binding and catalysis. Consequently, the simple conjugation of a fluorophore directly to the P1 position often results in a loss of enzymatic recognition.

**Scheme 5:**
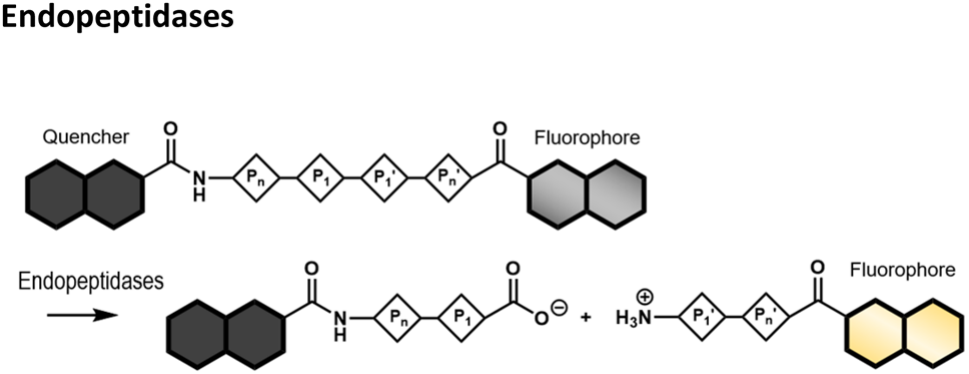
General design of endopeptidase probes.

To satisfy these recognition requirements, Förster resonance energy transfer (FRET)-based probes have become the primary architecture for endopeptidase activity sensing. These probes consist of a peptide linker containing the full recognition sequence, flanked by a donor fluorophore and a quencher (or an acceptor fluorophore) (Scheme 5). By placing the fluorophore and quencher at positions distal to the scissile bond (*e.g.*, at the N- and C-termini of a 5–10-mer peptide), the probe can maintain the necessary biochemical interactions within the enzyme's active site.^96^ Upon cleavage of the internal peptide bond, the donor and quencher are physically separated, leading to a dramatic increase in fluorescence intensity. The selective peptide sequence is primarily derived from the recognition sequence of the physiological substrate.^97^ The optimal distance between the fluorophore/quencher pair and the cleavage site is often a matter of empirical optimisation and differs among enzymes, depending on how deeply the peptide substrate penetrates into the active site. For example, in the case of MMPs, representative MMPs exhibited similar reactivity when residues distal to P5 and P2′ were modified,^96^ suggesting that the region between these positions is embedded within the enzyme active site.

**Fig. 13:**
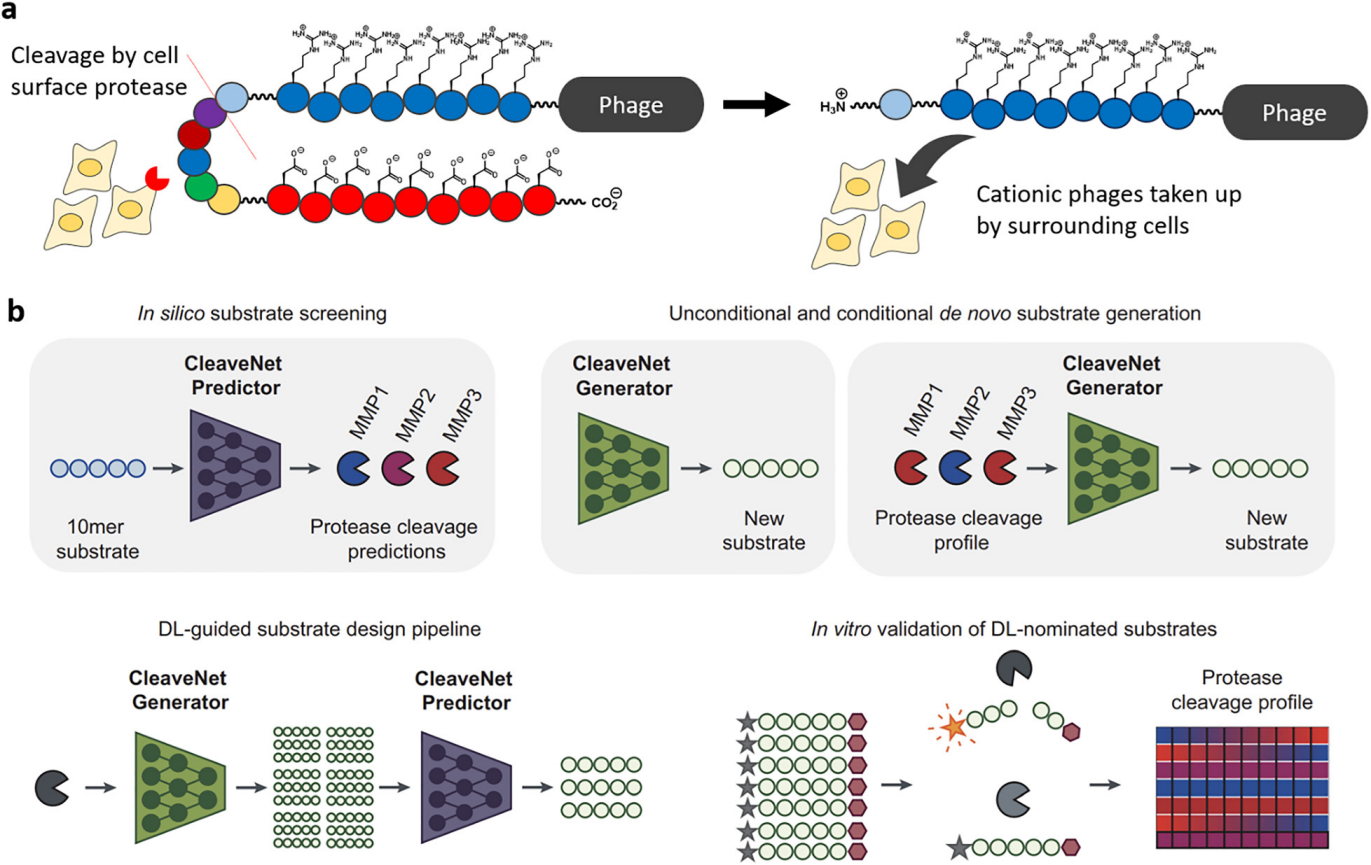
Design of selective reporter peptide for endopeptidases. (a) Phage-display-based selection of peptide sequences that are preferentially cleaved at tumour sites. Phages with the positively charged peptide, random sequences, and negatively charged peptides were introduced into cells/tissues. The cleavage of the random sequences in extracellular proteases expose the positively charged phages, that are taken up by surrounding cells.^94^ (b) The data science-based prediction of peptide sequences that are preferentially cleaved by the targeted proteases. Reproduced from ref. 95 with permission from Springer Nature, copyright 2026.

Attempts to perform rational substrate design are currently one of the major topics in this field. For example, phage display-based *in vivo* screening strategy reported by Whitney *et al.* revealed the peptide sequence that are preferably cleaved around tumour site and revealed the involvement of increased elastase activity in tumour regions^94^ (Fig. 13a). Recently, massive screening efforts combined with data science have enabled the prediction of selective sequences. Kukreja *et al.* reported a high-throughput multiplexed profiling of over 18 500 peptide sequences to determine the cleavage preferences of 18 MMPs, which led to the development of positional weight matrix (PWM)-based software capable of predicting cleavage sites with nearly 100% accuracy for certain enzymes.^98^ Ratnikov *et al.* utilized substrate phage display to screen approximately 64 million sequences, identifying specific residues surrounding the catalytic groove that serve as specificity-determining positions (SDPs), thereby providing a structural basis for the rational and focused redesign of cleavage specificity within the MMP family.^99^ Building on such large-scale datasets, Martin-Alonso *et al.* presented an AI-based pipeline called CleaveNet, which employs deep learning models to perform both *de novo* generation and selection of substrates tailored to specific protease activity profiles, demonstrating the ability to design synthetic substrates with exceptional selectivity for target enzymes such as MMP13^95^ (Fig. 13b). The selective sequence serves as the foundation for probe design in activity-based diagnostics using synthetic biomarkers,^4,100^ in which a substrate is administered and detection of the cleaved product is used as a readout of enzyme activity alterations associated with targeted diseases.

In designing FRET-based probes from the substrate peptide sequence, the N-terminus and C-terminus can be modified with either a fluorophore or a quencher. The choice is flexible, and in many cases, physicochemical properties and synthetic accessibility are considered. These physicochemical properties determine cell permeability and subcellular localisation, which are critical determinants of probe performance in live-cell or *in vivo* experiments. Myochin *et al.* reported that placing the fluorophore at the C-terminus is advantageous because the hydrolyzed product is efficiently internalized into cells, making this configuration suitable for live-cell staining.^101^ Ofori *et al.* also reported the value of positioning the fluorophore at the C-terminus, since the cleaved product, bearing a free amino group, tends to accumulate in lysosomes due to a lysosomotropic effect, making it suitable for fluorescence detection of cathepsin-overexpressing tumour cells in fluorescence-guided surgery.^102^

Alterations in endopeptidase activities have been widely reported in various diseases, including cancer and neurodegenerative disorders, and are frequently discussed in relation to physiological processes such as ageing and cell death. Recently, growing attention has also been focused on elucidating the roles of endopeptidases in immune system dynamics.^6^ Activated immune cells release specific endopeptidases such as granzymes and cathepsins in a cell-type-specific and condition-specific manner, making their enzymatic activity a powerful indicator of the immune response. For instance, granzyme B activity has been monitored using various activatable probes to evaluate T-cell-mediated cytotoxicity and predict the efficacy of cancer immunotherapies.^103–105^ Recently, Scott *et al.* have successfully utilized granzyme A-responsive probes to visualize adaptive immune cell function in real-time and monitor chronic inflammatory conditions.^106,107^

### Carboxypeptidases

Carboxypeptidases are exopeptidases that catalyse the hydrolysis of peptide bonds from the C-terminus of proteins and peptides. In the human genome, nearly 20 enzymes are considered to exhibit carboxypeptidase-type activities.^66^ Although the number is not high, they play important roles in regulating diverse biological processes, including digestive peptide processing, hormone maturation, and extracellular signalling. The C-terminal amino acid primarily determines the target enzyme class. Carboxypeptidase A (CPA) and related subtypes prefer aromatic residues at the C-terminus, whereas carboxypeptidase B (CPB) and related subtypes prefer basic amino acids at the C-terminus. Other subtypes, such as prostate specific membrane antigen (PSMA), prefer glutamic acid at the C-terminus.^66^

**Fig. 14:**
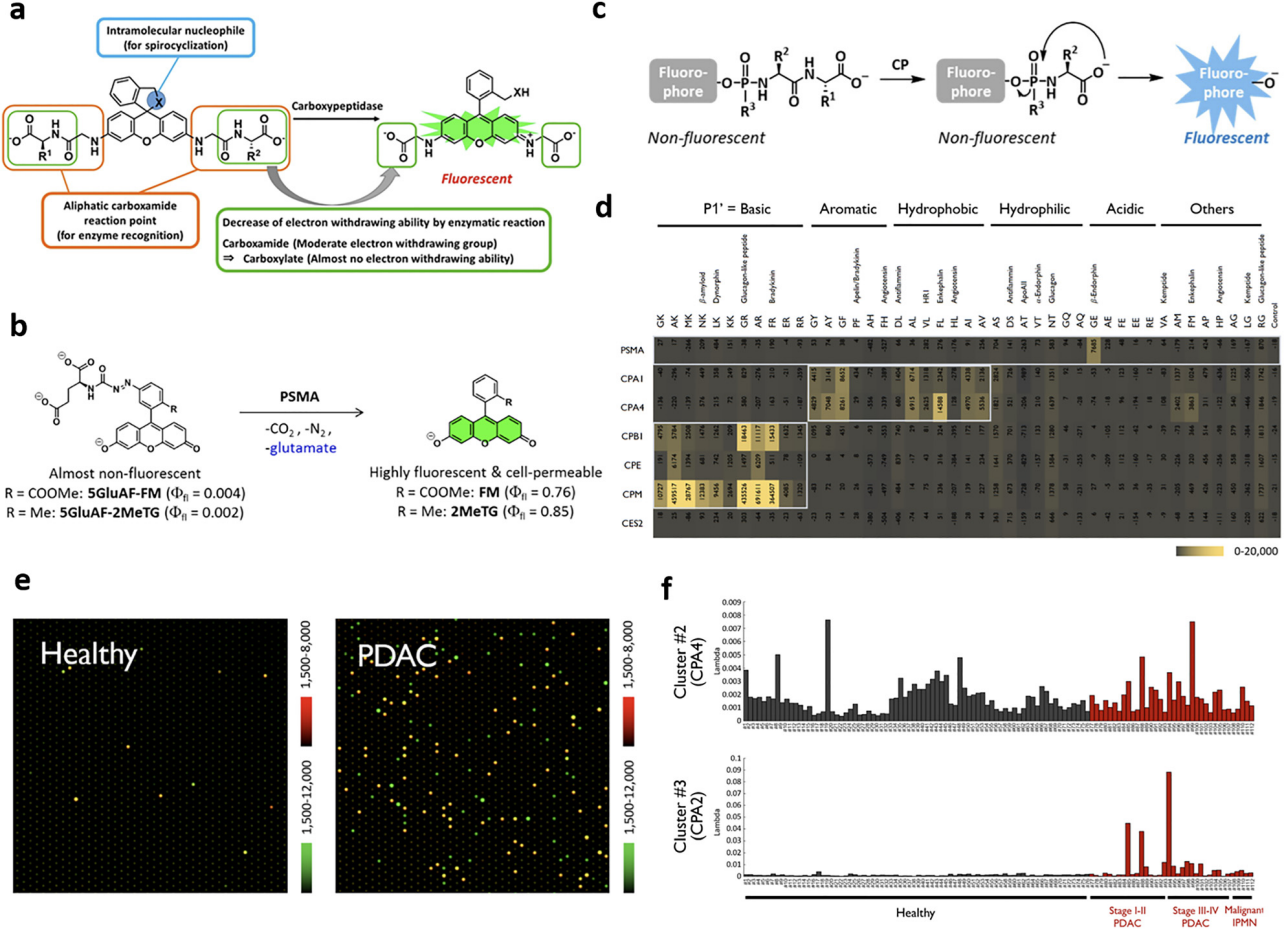
Design of carboxypeptidase probes. (a) Carboxypeptidase probe utilizing the changes of electron-withdrawing characters of amide and carboxylate. Reproduced from ref. 108 with permission from ACS, copyright 2018. (b) Carboxypeptidase probe utilizing azoformyl group. Reproduced from ref. 109 with permission from ACS, copyright 2019. (c) Design of carboxypeptidase probe utilizing ProTide chemistry. Reproduced from ref. 111 with permission from ACS, copyright 2024. (d) Profiling of carboxypeptidase substrate preferences using library of ProTide probes prepared by solid-phase synthesis. Reproduced from ref. 112 with permission from ACS, copyright 2026. (e) Single-molecule analysis of carboxypeptidases in blood samples of healthy subjects and PDAC patients. Reproduced from ref. 112 with permission from ACS, copyright 2026. (f) The activity of CPA4 (ubiquitous carboxypeptidase) and CPA2 (pancreas-specific carboxypeptidase) in blood samples of healthy subjects and PDAC patients. Reproduced from ref. 112 with permission from ACS, copyright 2026.

Despite their physiological importance, the development of activatable probes for carboxypeptidases has historically lagged behind that of aminopeptidases due to a fundamental lack of a generalisable design strategy. The defining feature of carboxypeptidases is their strict requirement for a free C-terminal carboxyl group for substrate binding. This poses a significant challenge for the “anilide-to-aniline” strategy. As an initial example, Kuriki *et al.* reported the design of translating the difference in electron-withdrawing properties between an amide and a carboxylate into a change in spirocyclization equilibrium, which is sensitive to inductive effects of amino group substituents^108^ (Fig. 14a). Another strategy is to utilize an azoformyl group that undergoes self-cleavage upon enzymatic reaction^109,110^ (Fig. 14b). However, these design strategies lack flexibility, making diversity-oriented synthesis highly challenging.

**Scheme 6:**
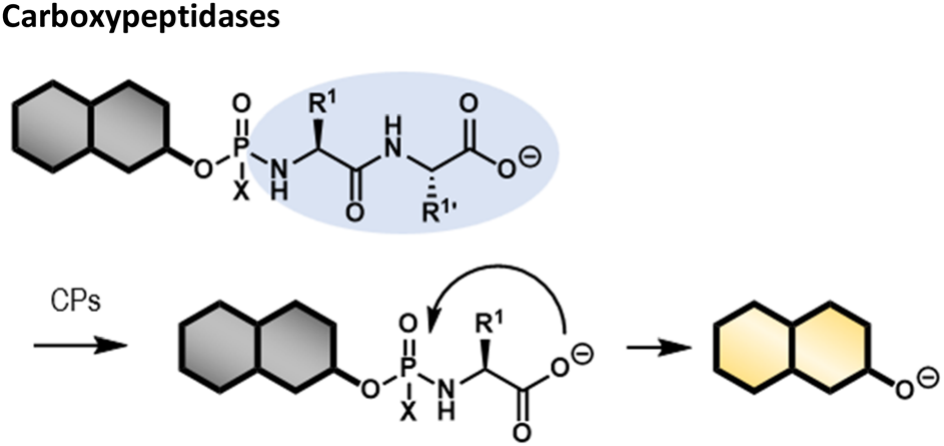
Design strategy of carboxypeptidase probes.

To overcome this obstacle, a new design rationale inspired by ProTide technology and self-immolative chemistry has recently been established by Kuriki *et al.*^111^ (Scheme 6 and Fig. 14c). Instead of using the C-terminal carboxyl group for conjugation, these advanced probes utilize a side-chain linkage or a self-immolative “masking” group that leaves the C-terminal carboxylate free and available for enzymatic recognition. This approach allows, for the first time, the sensitive and specific detection of carboxypeptidase activities such as CPA4 or carboxypeptidase R (CPR). The ability to design C-terminal-specific probes has opened new avenues in enzymomics. Solid-phase synthetic strategies for ProTide-based probes have been established,^112^ and substrate specificity profiling has revealed the key amino acid sequences that discriminate among diverse carboxypeptidase species (Fig. 14d).

One interesting feature of carboxypeptidases is that some enzyme species are expressed exclusively in specific tissues. For example, PSMA is predominantly expressed in the prostate,^113^ whereas CPA1, CPA2, and CPB1 are specifically expressed in the pancreas, and CPB2 is primarily expressed in the liver.^114^ Such tissue-restricted expression is not typically observed for aminopeptidases, whose expression patterns are rather ubiquitous; however, this feature is highly advantageous in biomarker discovery. Blood biomarkers are useful for disease detection because of their accessibility and low cost, but the difficulty in identifying the tissue of origin arises from the fact that blood contains proteins derived from multiple tissues. Therefore, alteration of a protein that is generated and released from several tissues may reflect pathological changes in different organs. In contrast, when expression is restricted to a specific tissue, the protein can serve as a highly specific biomarker of its tissue of origin.^112^ For example, PSMA is highly specific to the prostate and is therefore utilized as a biomarker for prostate diseases, including prostate cancer.^113^

In the similar fashion, pancreas-specific carboxypeptidases can serve as a good biomarkers of pancreatic disorders. Under the proposed concept of a “tissue-centric” biomarker discovery approach,^112,115^ a system to specifically monitor the activities of CPA1, CPA2, and CPB1 (pancreas-specific CP species) in blood samples was constructed, revealing that CPA1 and CPA2, whose expression is highly restricted to the pancreas, serve as specific biomarkers for pancreatic ductal adenocarcinoma (PDAC)^112^ (Fig. 14e).

### Dipeptidases and short peptide peptidases

**Scheme 7:**
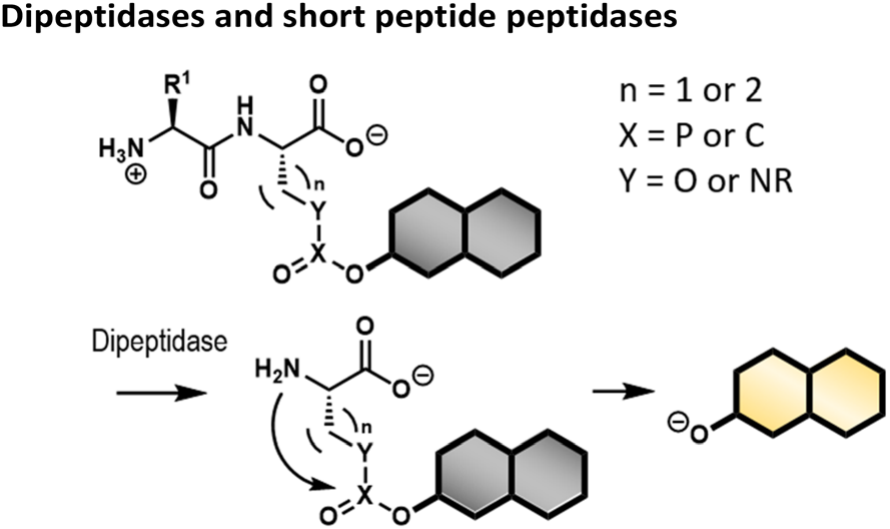
General design of short peptide peptidase probes.

**Fig. 15:**
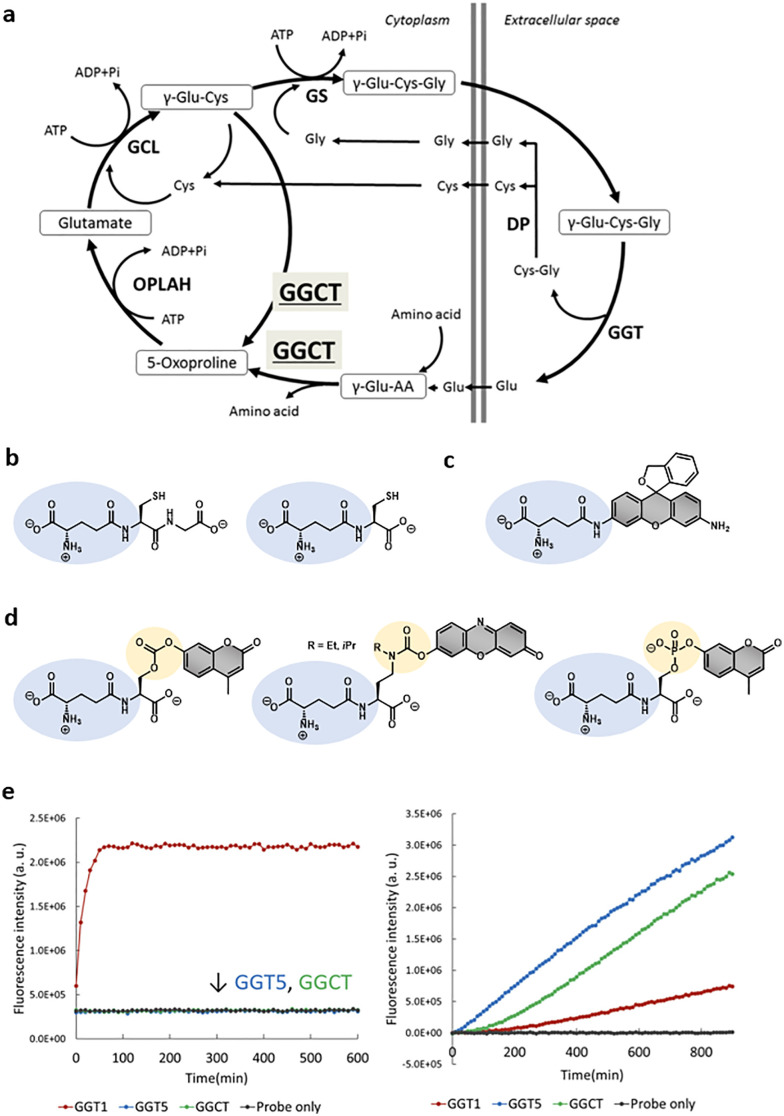
Design of fluorogenic probes for short peptide metabolising probes. (a) Glutathione cycle and enzymes responsible for the cycle. Reproduced from ref. 117 with permission from MDPI, copyright 2018. (b) Physiological substrates of GGT and GGCT. (c) Conventional fluorogenic probe for GGT1.^89^ (d) Fluorogenic probes with side-chain modification strategy, designed for GGCT.^116,118,119^ (e) Reactivity of GGT probe with direct modification of the fluorophore (left) and fluorogenic probe with side-chain modification strategy (right) against GGT1 (red), GGT5 (blue) and GGCT (green). Reproduced from ref. 119 with permission from ACS, copyright 2025.

These enzymes are challenging targets because they recognise both the N-terminal structure (the amino acid moiety in dipeptidases and the acylated amino group in acylamino acid hydrolases) and the C-terminal carboxyl group. As a result, it is difficult to introduce fluorophores at either terminus, and the design of FRET-type probes is also problematic. For these enzymes, an intramolecular reaction-based strategy has emerged as a practical solution (Scheme 7). In this approach, the side chain of an amino acid is modified with a fluorophore-containing group, and enzymatic cleavage triggers an intramolecular reaction that leads to fluorophore release.^116^ The original concept, proposed by Yoshiya *et al.*, was established for γ-glutamylcyclotransferase (GGCT), utilizing the free amino group generated after enzymatic reaction to attack an intramolecular carbonate moiety (Scheme 7). GGCT, alongside GGT, is a vital enzyme in the glutathione cycle, playing a critical role in maintaining cellular redox homeostasis and supporting cancer cell proliferation^117^ (Fig. 15a and b). This novel approach has enabled the visualization of enzymatic activities of GGCT that were previously undetectable with conventional fluorogenic substrates (Fig. 15c).

However, the ester-based strategy did not provide sufficient stability of the probe in the absence of enzyme activity. Therefore, an improved design employing carbamate^118^ or phosphate ester^119^ linkages was subsequently developed to enhance chemical stability while retaining enzymatic responsiveness (Fig. 15d and e). This strategy has been extended to targets including dipeptidases and acylamino acid hydrolases. Due to the limited availability of chemical tools, this class of enzymes has not yet been extensively explored in current biological studies. However, the development of new chemical probes is expected to shed light on these enzymes and clarify their biological significance.

Among peptidases and proteases that target long peptides or proteins, there exists a class of enzymes that recognise short peptide or amino acid derivatives, such as dipeptides or acylated amino acids. The former reaction is catalysed by dipeptidases, whereas the latter is catalysed by acylamino acid hydrolases.

### Esterase and lipases

Esterases have long been recognised as critical enzymes in pharmacology, playing a significant role in the metabolism of approximately 10% of clinical drugs.^120^ While their importance was historically captured through their capacity to detoxify or activate exogenous compounds, recent research has significantly advanced our understanding of their diverse endogenous roles, particularly in maintaining lipid homeostasis and regulating signalling pathways. Although esterases and lipases both belong to the serine hydrolase superfamily and share the ability to catalyse the hydrolysis of ester bonds, they are distinguished by their substrate preferences and structural characteristics.^120^ Esterases typically exhibit their highest activity toward water-soluble substrates and shorter-chain fatty acids. In contrast, lipases are characterised by their ability to act on aggregated or insoluble long-chain lipids at the lipid-water interface. This functional difference is often underpinned by a unique structural feature in many lipases known as the “lid” domain, which undergoes a conformational change to expose the active site and hydrophobic patches for substrate binding, a feature generally absent in most esterases.^120^

**Scheme 8:**
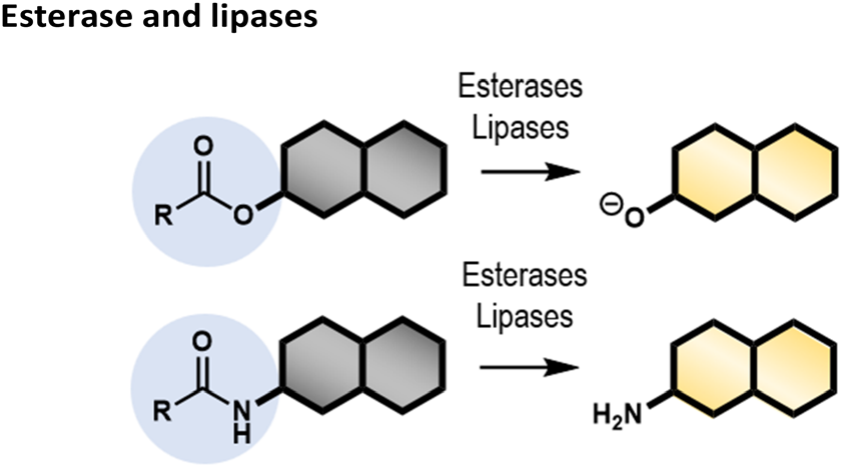
General design of esterase/lipase probes.

**Fig. 16:**
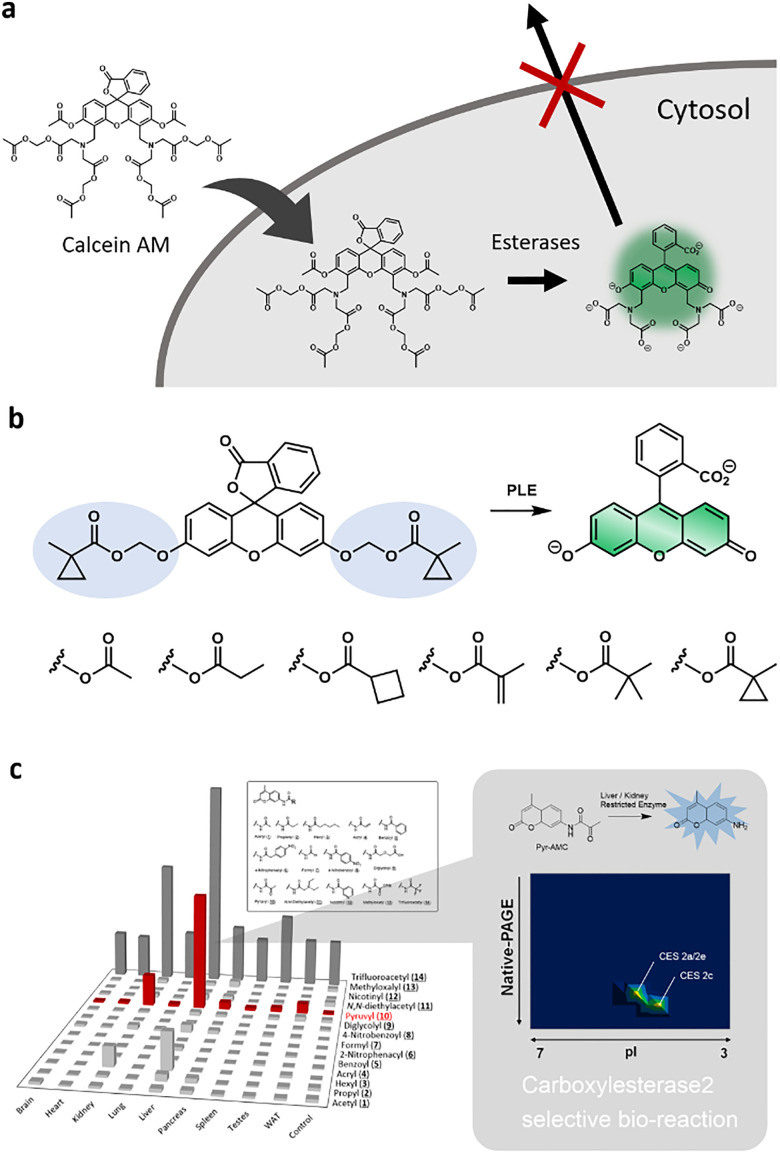
Various applications of esterase probes. (a) Intracellular uptake of calcein AM by live cells. (b) Optimisation of ester structure that shows selectivity toward porcine liver esterase (PLE).^123^ (c) Unexpected characterisation of metabolism of pyruvyl amide-type fluorogenic probe by CES2, a major subtype of carboxypeptidase. Reproduced from ref. 81 with permission from ACS, copyright 2015.

For esterases, a masking group strategy using various esters is commonly utilized (Scheme 8). Activity-based probes for esterases have traditionally been used for live-cell detection, such as fluorescein or calcein-based live-cell staining,^121,122^ because nonspecific ester hydrolysis is commonly observed in living biological samples (Fig. 16a). Simple ester moieties, such as acetyl or acetoxymethyl groups, have been widely utilized; however, Tian *et al.* demonstrated that incorporation of bulky acyl groups can impart subtype selectivity to specific esterases.^123^ The α-cyclopropyl group exhibited high specificity toward porcine liver esterase (PLE) and thus can be used as a reporter system (Fig. 16b). Such selective probe/substrate pairs have been employed as the basis for split-enzyme assays^124^ and for selective cellular targeting of functional probes.^125^

Although esterases were named for their high reactivity toward esters, their substrates are not strictly limited to esters, as certain amides can also be hydrolysed. An enzymomics study aimed at identifying tissue-specific amidase activities revealed that pyruvyl amide is selectively hydrolysed by carboxylesterase 2 (CES2) (Fig. 16c). This finding enables the design of fluorogenic probes based on an “amide-to-aniline” strategy.^81^ Efforts to develop fluorogenic probes for biologically important endogenous esterases and lipases generally follow a substrate-mimetic design strategy. Monoacylglycerol lipase (MAGL) is a representative example, playing a prominent role in the maintenance of endocannabinoid signalling and in tumour progression.^126^ Various fluorogenic probes for MAGL have been developed using an “ester-to-phenol” design, and these probes have been applied in high-throughput screening (HTS) and in studying activity alterations under different physiological conditions.^127^

**Scheme 9:**
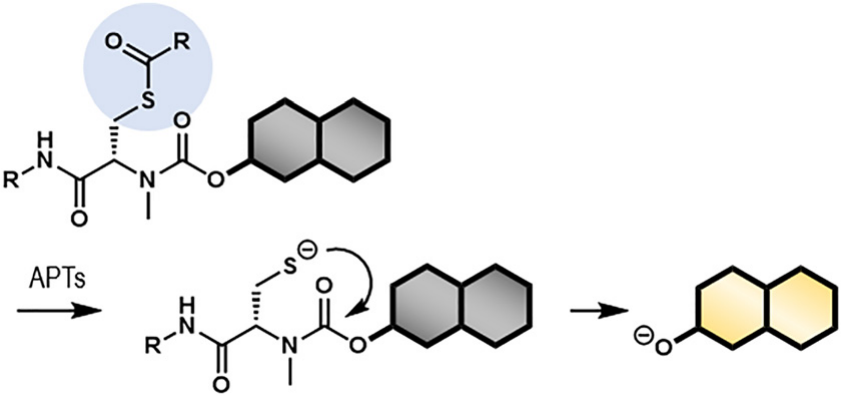
Design strategy of protein *S*-depalymitoylases.

In another example, fluorogenic probes for depalmitoylating enzymes (depalmitases) have been designed based on an intramolecular reaction mechanism.^128^ Depalmitoylating enzymes cleave the thioester bond of palmitate from cysteine residues. By incorporating an authentic cysteine thioester motif, probe activation was achieved through a thiol-transfer reaction. This thiol-transfer strategy is broadly applicable to the design of fluorogenic probes that monitor thiol-based post-translational modifications (PTMs) (Scheme 9).

**Fig. 17:**
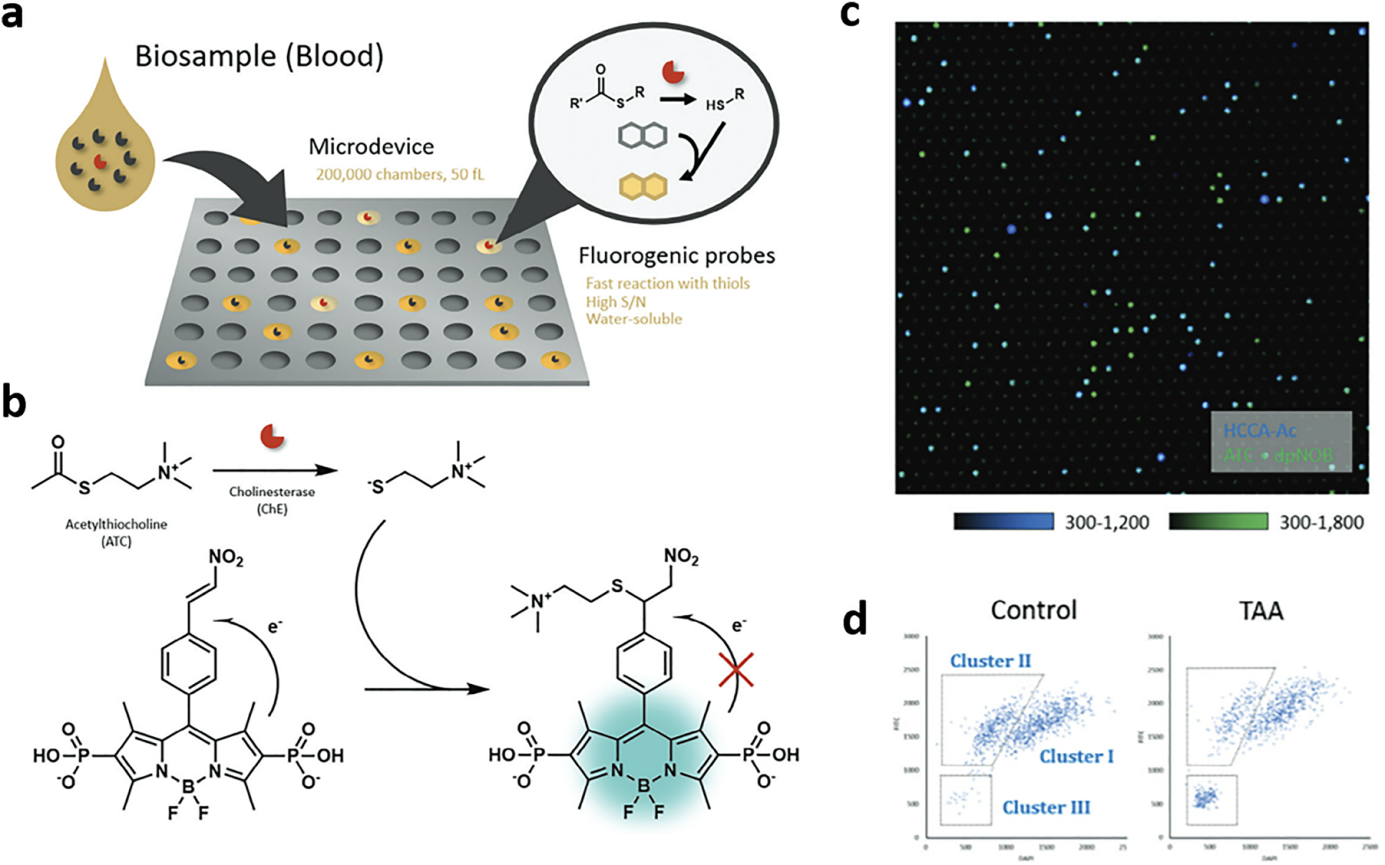
Design of single-molecule esterase activity analysis. Reproduced from ref. 129 with permission from Wiley-VCH, copyright 2024. (a) Design of thiol-based coupled assay to detect single-molecule enzyme activity assay of esterases. (b) Detection of cholinesterase activity using coupled assay. (c) Detection of single-molecule esterase activities in blood samples. (d) Alterations of enzyme activities in blood samples of control and liver damage (TAA) model.

In some enzymes, substrate recognition is so stringent that designing a fluorophore-conjugated substrate analogue becomes highly challenging. One representative example is acetylcholinesterase. While this enzyme exhibits a high catalytic turnover (*k*_cat_ = 6500 s^−1^), approaching the diffusion limit,^129^ it possesses extremely strict substrate specificity for acetylcholine. Therefore, the design of substrate analogues suitable for direct fluorogenic reporting is particularly difficult. For such enzymes, a coupled assay strategy—using the natural substrate or a close substrate mimic and detecting the product *via* an auxiliary detection system—is more appropriate. In the case of esterases, a thioester analogue is commonly employed. Because thioesters closely resemble their oxygen ester counterparts, enzymatic cleavage generates a free thiol, which can then be detected using thiol-selective fluorogenic probes. Following this coupled-assay strategy, Ukegawa *et al.* developed a single-molecule esterase activity assay, revealing the presence of multiple esterase species in blood samples and demonstrating activity alterations that correlate with liver damage^129^ (Fig. 17).

### Fluorogenic probes for oxidoreductases

Oxidoreductases are enzymes that catalyse oxidation–reduction reactions of substrates. According to enzyme classification by EC numbers, this class comprises the second largest group in terms of reaction types, following hydrolases. Because oxidative or reductive reactions usually do not involve direct bond cleavage, the general design of fluorogenic substrates is not straightforward. Nevertheless, intramolecular reaction-based strategies and coupled assay approaches have proven effective for certain subclasses of oxidoreductases.

Several oxidoreductases derived from microorganisms exhibit distinctive activities toward oxidative or toxic substrates, such as nitrobenzenes, azo dyes, and quinones. Because these substrates are not naturally occurring metabolites, such enzymes are thought to have evolved from oxidoreductases possessing broad substrate tolerance and high reductive capacity.^130^ A common feature of these enzymes is their use of nicotinamide adenine dinucleotide (and its phosphate form, NAD(P)H) as a reducing cofactor. Nitrobenzene, azobenzene, and quinone moieties have been primarily employed as targeting motifs for nitroreductases, azoreductases, and diaphorases, respectively, although overlapping activities among these enzyme classes have been observed in several cases.

### Nitroreductase (NTR)

**Scheme 10:**
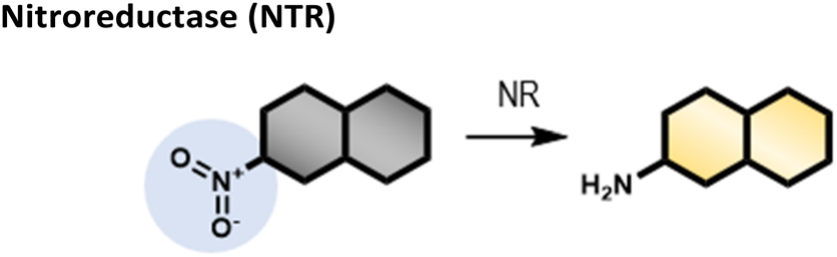
General design of nitroreductase probes.

Nitroreductases are a family of flavoproteins that utilize flavin mononucleotide (FMN) as a cofactor to catalyse the reduction of nitroaromatic compounds.^131^ The reaction typically follows a “ping-pong” mechanism, in which the bound FMN is first reduced by NAD(P)H, followed by the transfer of electrons to the nitro group (–NO_2_) of the substrate, reducing it to hydroxylamine or amine derivatives (Scheme 10). A primary application of NTRs is in cancer gene therapy, particularly in gene-directed enzyme prodrug therapy (GDEPT).^131^ Bacterial NTRs, such as those derived from *Escherichia coli*, efficiently activate the prodrug CB1954 into a potent DNA-crosslinking agent, far more effectively than endogenous human enzymes.^131^ This selective activation underlies their therapeutic utility. In addition, NTRs are increasingly employed in noninvasive *in vivo* imaging applications. Quenched fluorogenic probes, such as CytoCy5S, function as substrates that become highly fluorescent upon enzymatic reduction by NTR, thereby enabling spatiotemporal monitoring of processes such as metastatic cancer progression.^132^

### Azoreductases

**Scheme 11:**
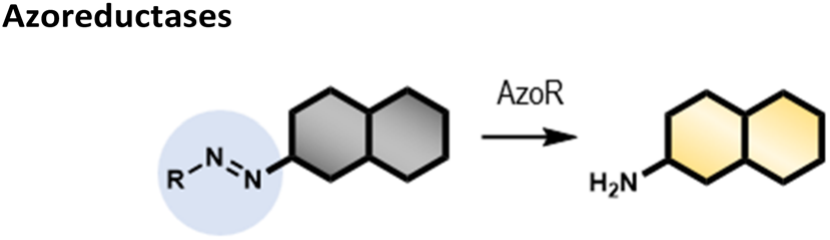
General design of azoreductase probes.

**Fig. 18:**
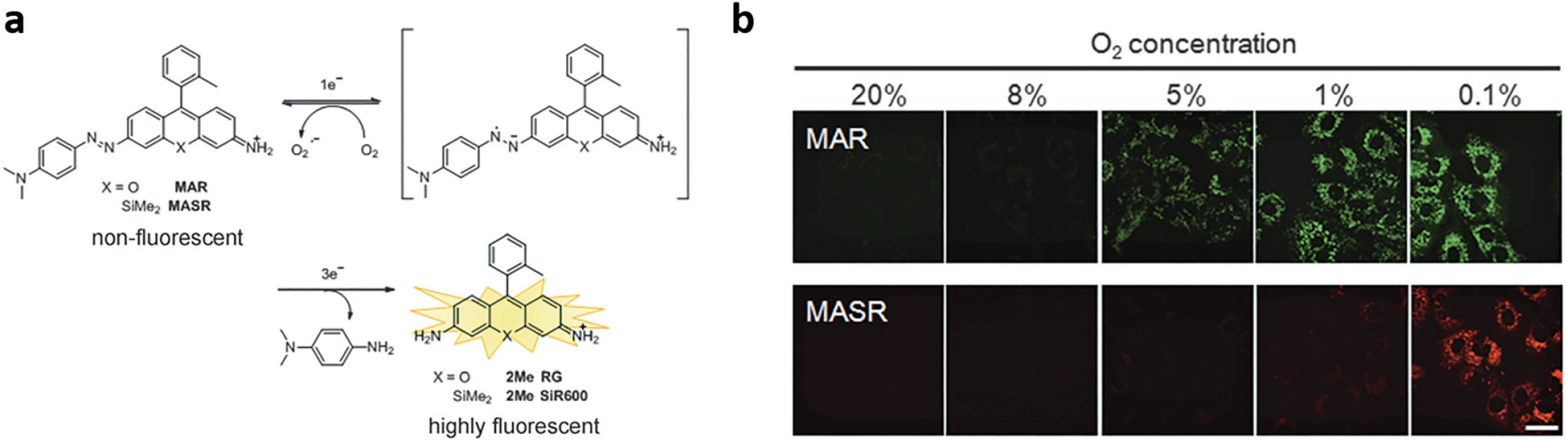
Design of fluorogenic probes responding to hypoxia. Derived from ref. 133 with permission from Wiley-VCH, copyright 2013. (a) Structures of azo-baring fluorogenic probes that reacts with intracellular azo-reductases for fluorescence activation under hypoxic conditions. (b) Detection of different levels of O_2_ using different azoreductase probes.

Azoreductases are oxidoreductases that catalyse the reductive cleavage of the azo bond (–N=N–), a characteristic functional group found in various synthetic dyes and select natural compounds.^130,134^ The enzyme utilizes two equivalents of NAD(P)H and two protons to reduce one equivalent of an azo compound into two separate aniline derivatives (Scheme 11). Similar to nitroreductases, the reaction proceeds *via* a ping-pong mechanism. In mammalian cells, azo compounds are preferentially reduced under hypoxic (low O_2_) conditions, because molecular oxygen competes with the reduced flavin intermediate. Therefore, azobenzene-containing probes have been widely used to monitor and visualize hypoxic environments^133,135–138^ (Fig. 18). Interestingly, the dependence on O_2_ concentration varies according to the reduction potential of the azo substrate, enabling the design of probes with tunable hypoxia sensitivity.^133,138^ This property has been exploited to monitor gradients of O_2_ concentration within hypoxic tumour tissues (Fig. 18b).

### Diaphorase (NADPH dehydrogenase)

**Scheme 12:**
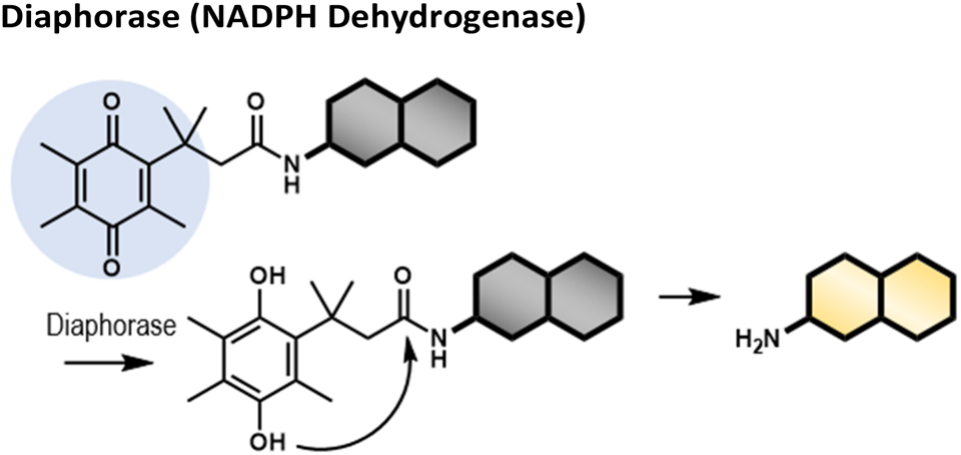
General design of diaphorase probes.

Diaphorase, primarily classified as NADPH dehydrogenase, is a flavoprotein that facilitates electron transfer from NADPH to a wide variety of acceptors. Historically referred to as the “old yellow enzyme,” it typically contains flavin cofactors such as FMN or FAD. The general reaction involves oxidation of NAD(P)H to NAD(P)^+^ concomitant with reduction of an external electron acceptor. Unlike the more specialized nitroreductases or azoreductases, diaphorases exhibit broad substrate promiscuity and can act on diverse acceptors. Among these, quinone-based substrates—particularly those bearing a quinone propionic acid moiety—have frequently been employed as optimal trigger structures (Scheme 12). Upon reduction by diaphorase, the resulting hydroquinone undergoes an intramolecular cyclization, in which a phenolic group attacks the adjacent amide bond to release the free fluorophore.^12^ By utilizing quinone propionic acid as a trigger moiety, fluorogenic probes selectively activated by NAD(P)H:quinone oxidoreductase 1 (NQO1) in mammalian cells have been developed.^139^ These probes have been successfully applied to *in cellulo* and *in vivo* imaging of cancers in which hNQO1 is highly upregulated, including colorectal, lung, and ovarian cancers.^12^

**Fig. 19:**
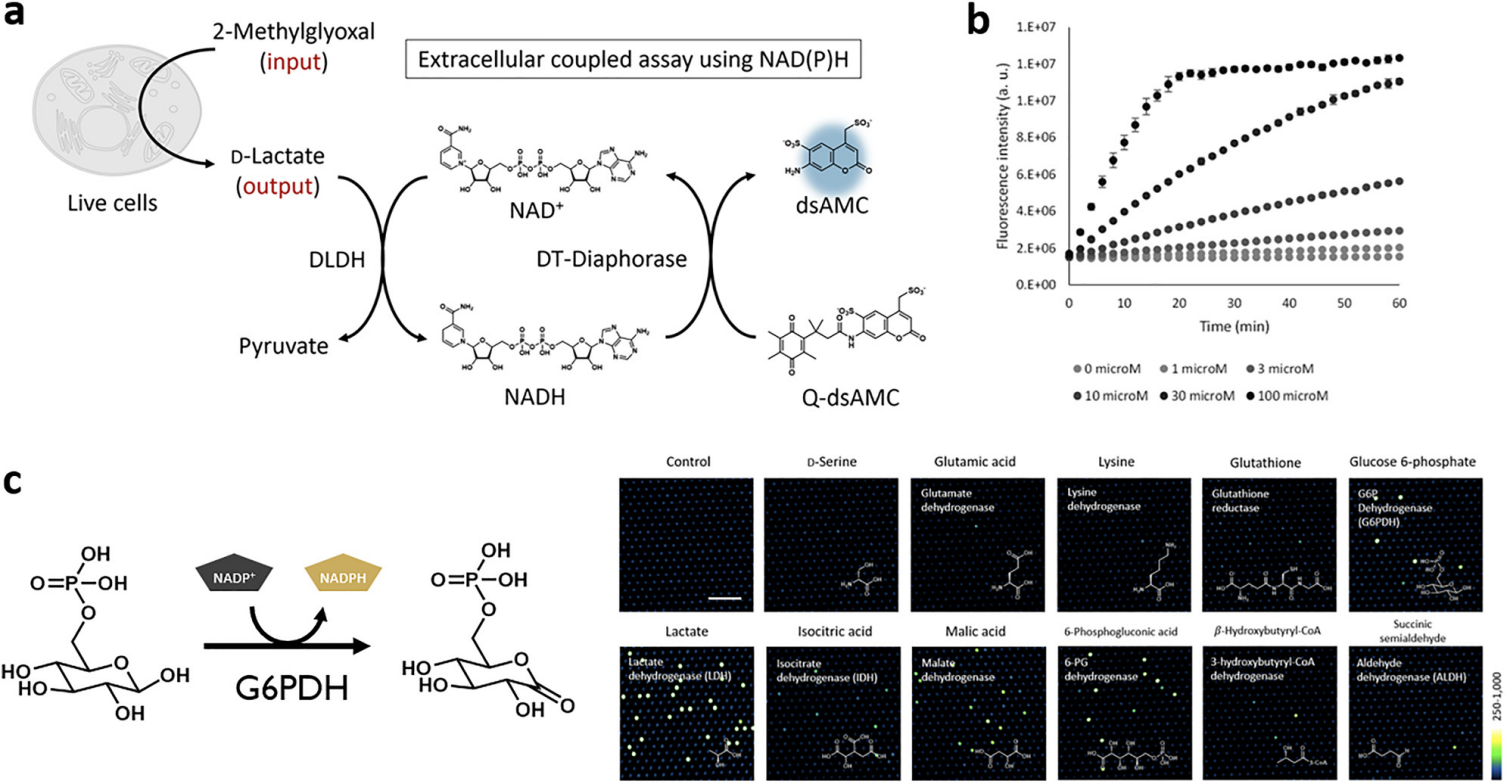
Fluorescence detection of diaphorase for coupled assay of NAD(P)H. (a) Detection of live cell metabolism using extracellular coupled assay. Derived from ref. 143 with permission from Springer Nature, copyright 2023. (b) Response of fluorogenic probe in (a) for detection of different concentrations of d-lactate using d-lactate dehydrogenase. Derived from ref. 143 with permission from Springer Nature, copyright 2023. (c) Detection of G6PDH and other oxidoreductases in single-molecule enzyme activity assay platform. Derived from ref. 140 with permission from ACS, copyright 2025.

Using recombinant diaphorase, the reaction can proceed nearly quantitatively in proportion to the concentration of NAD(P)H; thus, this system is also widely utilized for NAD(P)H detection (Fig. 19a and b). Because more than half of oxidoreductase enzymes employ NAD(P)^+^/NAD(P)H as redox cofactors,^140^ the activities of other oxidoreductases can be conveniently monitored through coupling with this reaction. Traditionally, small-molecule-metabolising enzymes have been linked to NAD(P)^+^/NAD(P)H-converting reactions *via* appropriate dehydrogenases, and NAD(P)H has been detected using the formazan/tetrazolium assay, in which formation of a coloured tetrazolium salt serves as the readout.^141^ However, the diaphorase-based reaction is rapid and proceeds in an almost quantitative manner, making it particularly suitable for the design of sensitive coupled assays involving NAD(P)H to monitor the activities of small-molecule metabolisingactivity of cells in live-cell mode or at single-molecule level^140,142,143^ (Fig. 19c).

### Monoamine oxidase

Oxidases constitute another major class of oxidoreductases, catalysing the oxidation of substrates with molecular oxygen to generate H_2_O_2_ as a byproduct. Because many oxidases act on small-molecule metabolites, the design of fluorophore-conjugated substrate analogues is often challenging. Therefore, their activities have traditionally been analysed indirectly through detection of H_2_O_2_. Nevertheless, several specific oxidases can be investigated using appropriately designed reaction-based probes.^141^

**Scheme 13:**
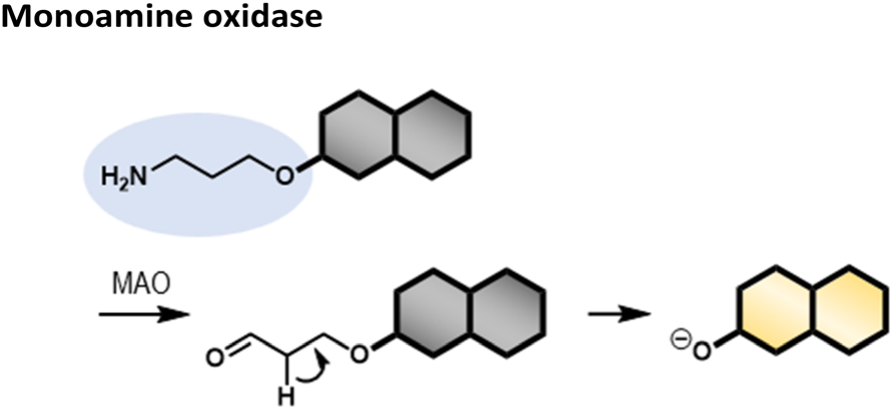
Design strategy of monoamine oxidase probes.

Monoamine oxidases (MAOs) catalyse the oxidation of alkylamines to the corresponding aldehydes. Exploiting the formation of aldehydes as reactive intermediates, fluorogenic strategies have been developed to monitor MAO activity. Initially, Chen *et al.* reported a probe design based on an intramolecular cyclization reaction.^144^ However, limited generality left room for further optimisation. Subsequently, a more versatile enzyme–substrate system was introduced by Albers *et al.*, who employed a β-elimination reaction of 4-phenoxypropylaldehyde^145^ (Scheme 13). In this design, the propylamine moiety was identified as a broadly reactive motif targeting monoamine oxidases.

Efforts to control subtype selectivity and to expand applications to sophisticated *in cellulo* and *in vivo* imaging have since been reported. Nonetheless, the fundamental reaction principle—aldehyde-triggered β-elimination following MAO-mediated oxidation—remains a core strategy for designing activity-based probes targeting monoamine oxidases.^10,11,13^

### Cytochrome P450

**Scheme 14:**
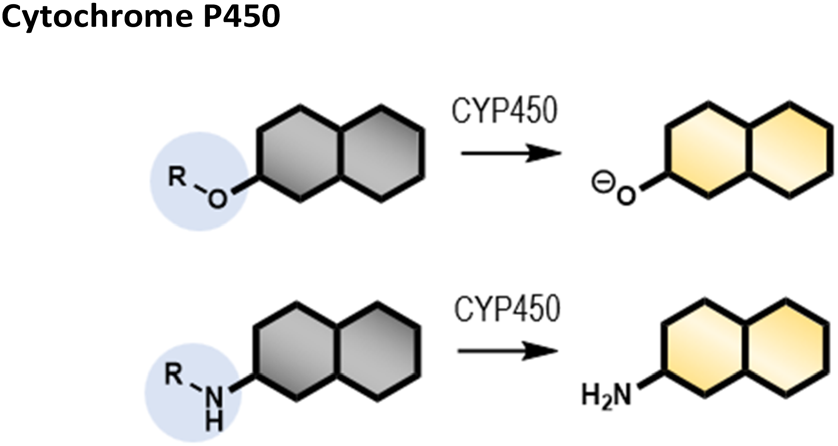
Representative reactions used to monitor the activity of cytochrome P450.

**Fig. 20:**
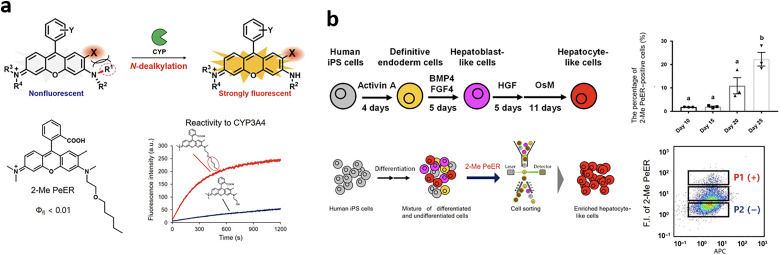
Applications of CYP450-reactive fluorogenic probes. Derived from ref. 146 with permission from AAAS, copyright 2024. (a) Molecular design of CYP450-reactive fluorogenic probes. (b) Real-time imaging of hepatocellular cell differentiation by monitoring the expression of CYP3A4. (c) FACS-based sorting of iPS-derived hepatocellular cells using CYP3A4-reactive fluorogenic probes.

Cytochrome P450 enzymes are among the most important catalysts involved in drug metabolism. They are highly expressed in the liver, where they play a central role in detoxifying hydrophobic compounds by metabolically introducing polar functional groups, such as hydroxyl moieties, thereby increasing hydrophilicity and facilitating excretion (Scheme 14). The unique catalytic mechanism of P450 enzymes, together with overlapping substrate recognition among diverse CYP isoforms, makes the design of isoform-selective fluorogenic substrates particularly challenging. Nevertheless, 7-ethoxycoumarin has been widely used as a representative fluorogenic substrate, as its *O*-dealkylation is catalysed by P450 enzymes, especially CYP1A1 and related CYP1A subtypes. The reaction generates a fluorescent product, providing a convenient readout of enzymatic activity. More recently, selective detection of *N*-dealkylation catalysed by CYP3A4 has been achieved using tailored fluorogenic probes. This strategy has been applied to fluorescence-activated cell sorting (FACS)-based analyses of cell differentiation, in which CYP3A4 expression serves as a functional marker for the differentiation of induced pluripotent stem (iPS) cells into hepatocyte-like cells^146^ (Fig. 20).

In addition to probes for monitoring CYP450 catalytic turnover, the oxidative mechanism characteristic of the P450 cycle^147^ has been chemically adapted to develop sensors for its cofactor; for instance, Hirayama *et al.* reported an *N*-oxide-driven labelling strategy to map the dynamics of labile heme itself *via* ferryl-oxo intermediates.^148^

## Other design strategies of fluorogenic probes

While we have discussed the major enzyme/substrate pairs used in designing fluorogenic substrates, they do not fully capture the diversity of the enzymome, and many additional enzymes remain attractive targets for visualization. Two particularly challenging yet promising categories are post-translational modification (PTM) enzymes and enzymes metabolising small-molecular metabolites.

### PTM enzymes

**Scheme 15:**
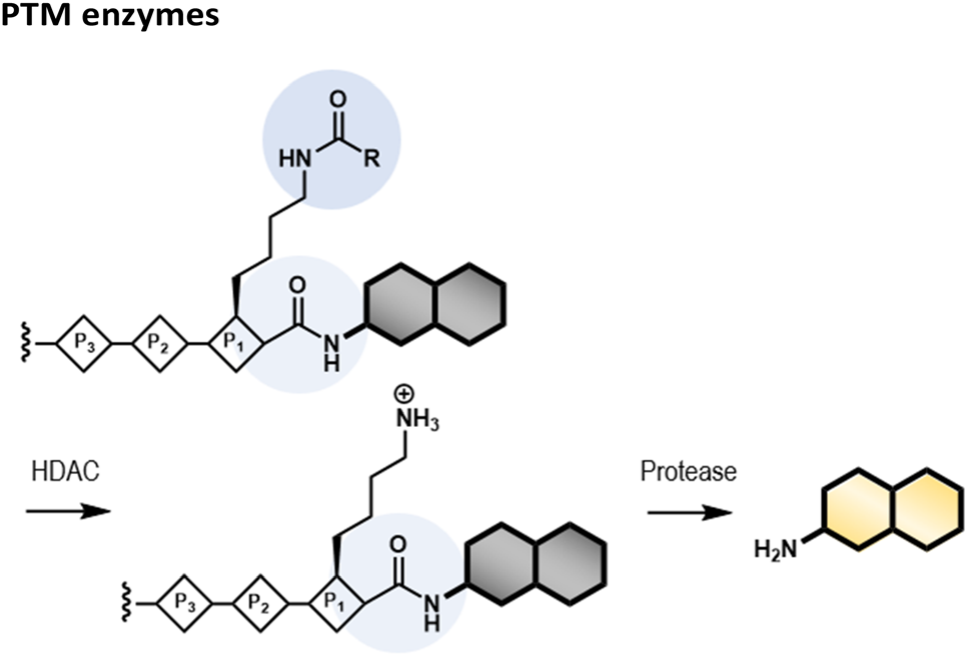
Design of coupled assay probe for PTM enzymes having lysine PTM-modulating enzymes as an example.

Protein function is regulated by a wide variety of post-translational modifications, and more than 200 distinct PTMs have been identified. Peptides provide an excellent platform for monitoring these modifications, as they can mimic protein substrates while incorporating fluorescence-based detection systems. However, converting specific PTMs—other than hydrolytic events—into fluorescence changes is generally challenging. The traditional solution has been the use of coupled assays. The general concept is to leverage the high sequence specificity of certain proteases and design substrates in which protease recognition changes dramatically depending on the presence or absence of a targeted PTM, thereby converting PTM activity into a measurable proteolytic event (Scheme 15).

**Fig. 21:**
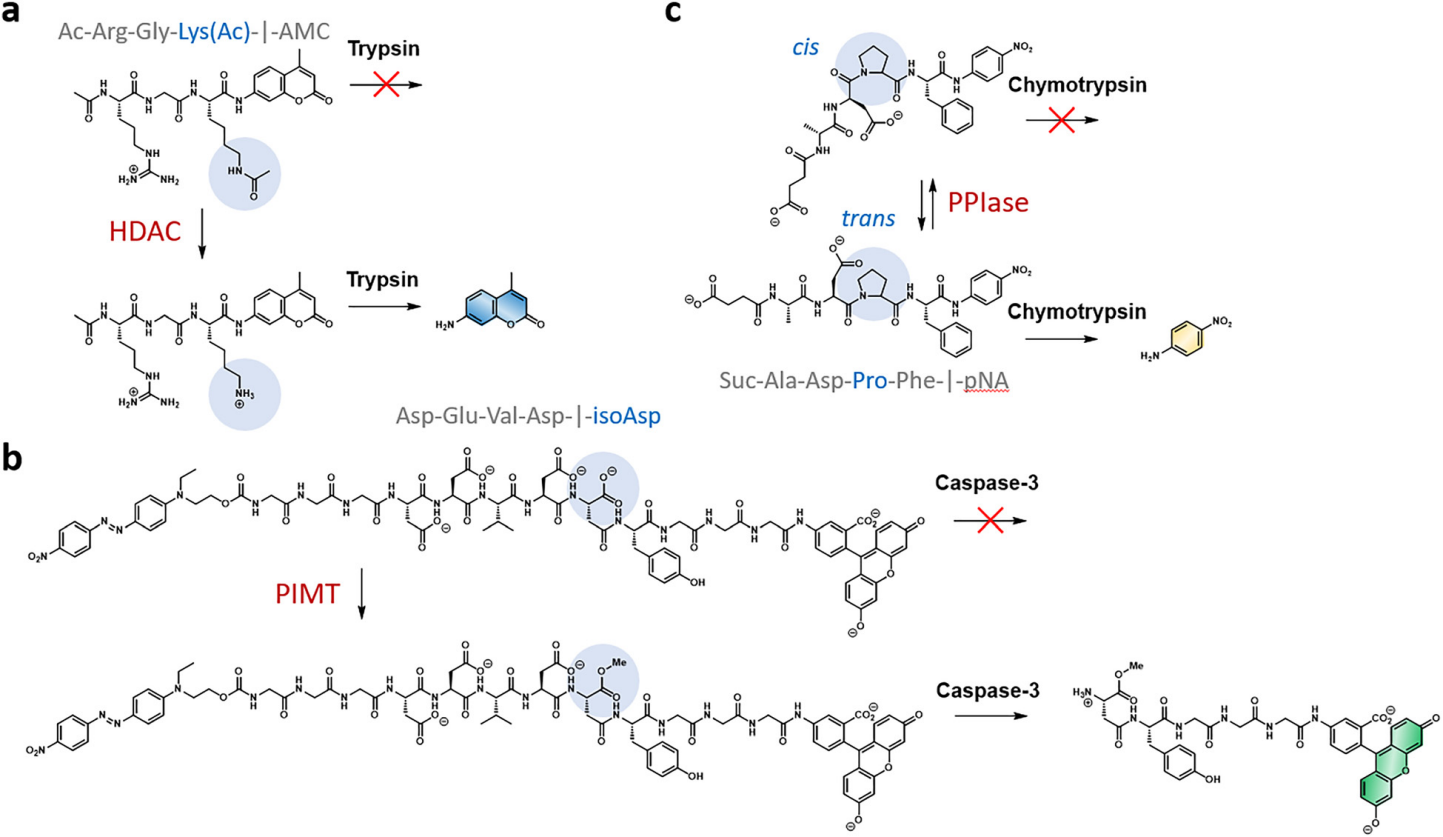
Design of coupled assays for various PTM enzymes. (a) Coupled assay probe for HDAC coupled with trypsin.^149^ (b) Coupled assay probe for protein l-isoaspartyl transferase (PIMT) coupled with caspase-3.^150^ (c) Coupled assay probe for proline *cis*/*trans* isomerase (PPIase) coupled with chymotrypsin.^151^

Histone deacetylases (HDACs) are critical enzymatic regulators that remove acetyl groups from lysine residues, playing essential roles in epigenetic regulation and serving as key therapeutic targets for cancer and neurodegenerative diseases. Traditional coupled assays exploit the fact that acetylated lysine residues inhibit proteases such as trypsin; once the acetyl group is removed by HDAC, the coupled protease cleaves the peptide to release a C-terminal fluorophore^149,152^ (Fig. 21a). Beyond these enzymatic coupling strategies, non-enzymatic coupled systems have also been developed, in which the primary amine generated by deacetylation acts as an intramolecular nucleophile, triggering spontaneous transesterification and restoring coumarin fluorescence without requiring an additional protease.^153^ Furthermore, for Sirtuins, FRET-based probes have been designed to exploit their ability to recognise long-chain fatty acyl groups; in such systems, SIRT-mediated hydrolysis removes a bulky Dabcyl quencher attached to the lysine side chain, thereby restoring fluorescence.^154,155^

Although lysine methylation has been extensively studied using fluorogenic probes, many other PTMs remain less explored. For example, protein l-isoaspartyl methyltransferase (PIMT) is a repair enzyme that specifically methylates isoaspartyl residues formed through spontaneous protein damage, a process associated with diseases such as epilepsy and cancer.^156^ Despite its biological importance, methods for monitoring its activity are still limited. Kimura *et al.* proposed a substrate design in which an isoaspartyl residue is placed at the P1′ position of a caspase-3 recognition sequence.^150,157^ The negative charge of the isoaspartyl residue suppresses Caspase-3 activity; after methylation by PIMT, the charge is masked, enabling caspase-3 cleavage and subsequent fluorescence activation (Fig. 21b).

Peptidyl prolyl *cis*–*trans* isomerases represent another biologically important but underexplored enzyme class. More than 20 enzymes belong to this family, yet successful activity-monitoring strategies remain limited. These enzymes regulate protein folding and function by catalysing proline peptide bond isomerization. Well-characterised members, such as FKBP and Pin1, are implicated in immune and neurodegenerative diseases.^158,159^ Traditionally, their activities are monitored using chymotrypsin, which selectively cleaves the *trans* isomer of proline-containing substrates to release chromophores such as 4-nitroanilide^160^ (Fig. 21c). Although this classical assay remains widely used, it is often limited by complex biphasic kinetics and insufficient precision, underscoring the need for more accurate and direct assay systems.

### Enzymes metabolising small molecular metabolites

Another challenging class in enzyme activity monitoring comprises enzymes that metabolize small molecules, introducing subtle structural changes. These enzymes operate in central metabolic pathways, converting glucose, amino acids, and lipids into energy and biosynthetic building blocks. Designing fluorogenic switches for such transformations is inherently difficult; nevertheless, pioneering studies have demonstrated promising strategies.

**Fig. 22:**
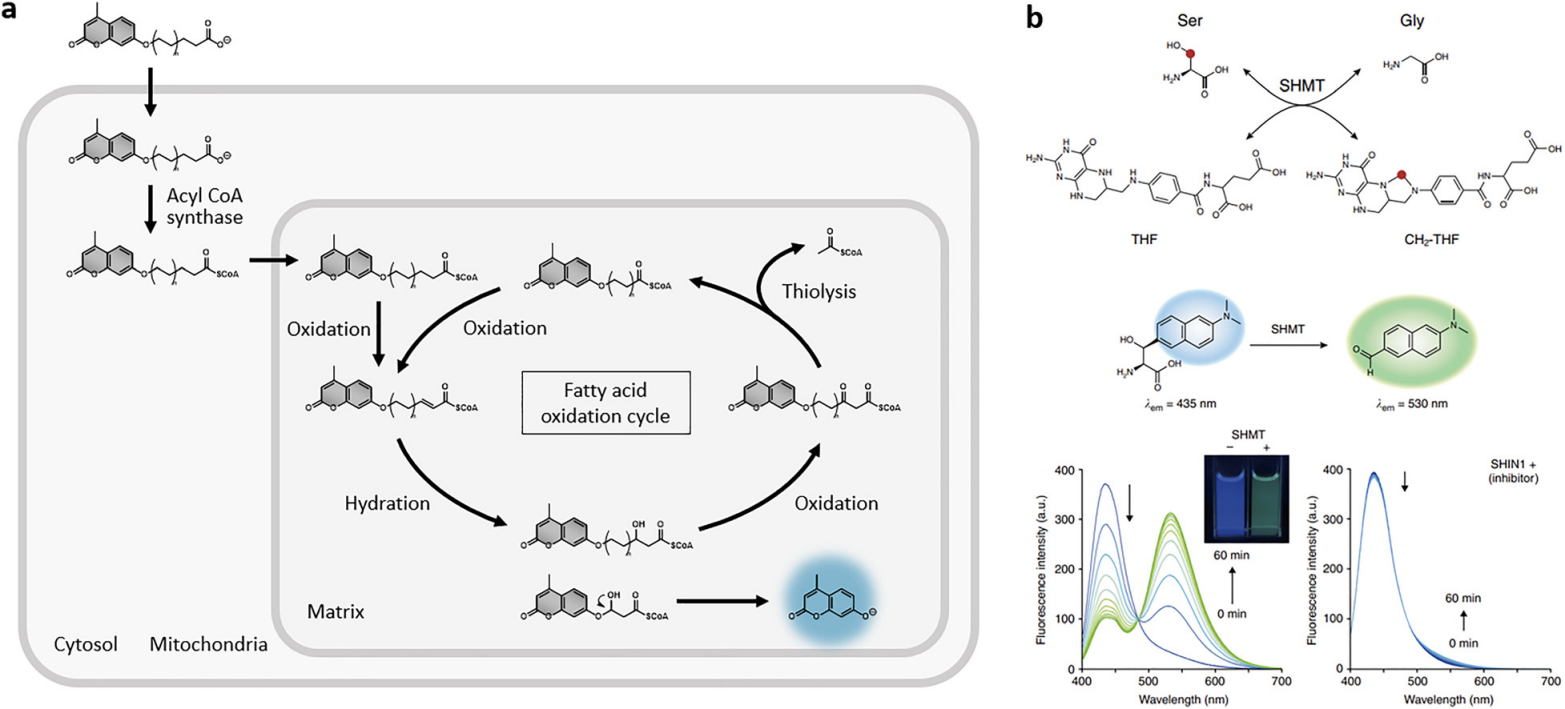
Design of fluorogenic probes targeting enzymes metabolising small-molecular metabolites. (a) Design of fluorogenic probe to monitor fatty acid oxidation (β oxidation) cycle.^161^ (b) Fluorogenic probe that are designed to act as a substrate mimic of serine hydroxymethyltransferase (SHMT).^162^ Reproduced from ref. 162 with permission from Springer Nature, copyright 2019.

Uchinomiya *et al.* designed lipid-modified fluorogenic probes to monitor β-oxidation activity in living cells.^161,163^ The probe consists of a fatty acid moiety conjugated to a coumarin fluorophore, which is transported into mitochondria *via* the carnitine shuttle. It undergoes sequential degradation through the fatty acid oxidation (FAO) cycle, ultimately releasing bright 7-hydroxycoumarin *via* spontaneous hydrolysis of a hemiacetal intermediate (Fig. 22a). This innovative “turn-on” design enabled real-time monitoring of FAO flux in individual living cells for the first time.

In another example, Nonaka *et al.* developed a fluorogenic substrate for serine hydroxymethyltransferase (SHMT).^162^ By focusing on a tetrahydrofolate (THF)-independent retro-aldol reaction rather than the canonical serine–glycine interconversion, they overcame spatial constraints within the enzyme's active site. The probe incorporates a reporter group at the β-position of serine, which is enzymatically converted into an aromatic aldehyde. This transformation induces a significant ratiometric fluorescence change, providing a direct and sensitive method for detecting SHMT activity even in complex biological samples (Fig. 22b). These examples illustrate how the design of enzyme-targeting fluorogenic probes constitutes a central pillar of chemical biology, with successful developments reflecting the creativity of reaction design. It is hoped that this collection of pioneering strategies will inspire further advances in the development of fluorogenic probes for biologically important yet still underexplored enzymes.

## Conclusion

“Revisiting the past to discover the new” is a traditional saying in East Asian culture that emphasizes extracting profound insights from historical knowledge to inform contemporary understanding. The chemical design of fluorogenic substrate analogues—exemplified by the classic “ester-to-phenol” and “amide-to-aniline” strategies—has a history spanning more than a century. While the mechanisms of fluorescence control and their biological applications have evolved significantly, enabling increasingly precise evaluation of enzymatic activities, the fundamental chemistry continues to be grounded in these foundational design principles.

Advances in substrate design and activity-monitoring platforms have led to a better understanding of the biological roles of enzymes and their contributions to physiological processes and diseases. Furthermore, deep chemical insights into enzymatic mechanisms often inspire developments in the broader field of activity-based sensing (ABS),^147,148^ where the synergy between fundamental biochemistry and innovative molecular design continues to expand the chemical toolbox for a better understanding of the functional complexity of life.

Over the past hundred years, our understanding of proteins has advanced remarkably, largely driven by progress in molecular and cell biology. However, the prevailing molecular biology paradigm—often centred on understanding biological function on a “per-gene” basis—faces inherent limitations. First, it frequently overlooks the dynamic nature of protein “activity,” which does not necessarily correlate with protein “abundance”.^9^ Second, it struggles to account for the extensive diversity of the proteome, shaped by post-translational modifications and protein–protein interactions, which extends far beyond the classical “one gene, one protein” framework.^8,164,165^

From this perspective, the power of chemical tools to interrogate protein function is being reappreciated as a means to reveal their true contributions to phenotypic changes and disease states.^7^ This renewed emphasis opens a new horizon for enzyme-responsive chemical probes, positioning them as indispensable tools for deciphering the functional complexity of the active proteome in the modern era.

## Author contributions

T. K. and Y. U. wrote the manuscript.

## Conflicts of interest

T. K. is a shareholder and advisor of Cosomil, Inc.

## Data Availability

No new data were created or analyzed in this study. Data sharing is not applicable to this article.
